# Inter- vs. Intra-Speaker Variation in Mixed Heritage Syntax: A Statistical Analysis

**DOI:** 10.3389/fpsyg.2019.01528

**Published:** 2019-07-11

**Authors:** Federica Cognola, Ivano Baronchelli, Evelina Molinari

**Affiliations:** ^1^Dipartimento di Studi Europei, Americani e Interculturali, Sapienza Università di Roma, Rome, Italy; ^2^Dipartimento di Fisica e Astronomia, Università di Padova, Padua, Italy; ^3^Istituto Culturale Mòcheno, Palù del Fersina, Italy

**Keywords:** word order, OV/VO, negative concord, scrambling, information structure, intra-speaker variation, inter-speaker variation, double-base hypothesis

## Abstract

Based on the novel data pertaining to five syntactic phenomena (the position of the finite verb in embedded clauses, in sentences with a modal verb, negative concord, the position of focused light/heavy objects in main clauses with a complex tense and scrambling) in the heritage language Mòcheno collected via original fieldwork, we show that there are two populations – one exhibiting intra-speaker variation between German and Italian word orders, and one lacking it; and these two populations are the result of diatopic variation and, to a lesser extent, of diastratic variation. The results achieved using quantitative statistical analysis are partially convergent with those arrived at via the traditional theoretical syntax for Mòcheno, but our analysis has allowed us to shed new light on a series of phenomena that have been neglected or poorly understood thus far. More specifically, and for the first time, we discovered that there is a micro-variation resulting from diastratic (age) variation within the Roveda variety, which represents the only case in Mòcheno in which age is a relevant factor in determining variation. We also show that the traditional claim that the Palù variety is ‘more German’ than is the other Mòcheno variety is to be confirmed; however, we could refine it by showing that German word orders are also accepted by speakers of other varieties and that the acceptability of these word orders in competition with the Italian syntax is not due to their age (no diastratic variation). Finally, we show that the acceptance of German word orders across speakers varies according to the phenomenon investigated: German word orders are more likely to be accepted in sentences featuring a negation, whereas German word orders are more likely to be rejected in embedded clauses. Based on this fine-grained description of the distribution of OV/VO word orders across different contexts and groups and the available theoretical account for the derivation of OV word order given by Cognola (2013b), we propose that the observed variation can be parametrized along the lines of recent developments of Parameter Theory (Roberts, 2012; Biberauer et al., 2014 a.o.). More specifically, we propose that the movement of the non-finite verb form to lowForce°, which is responsible for OV in Mòcheno, can be captured in terms of a parametric hierarchy. When verb movement takes place in all syntactic conditions, including with all non-finite verb forms and when the auxiliary has not moved out of v° to Spec, CP, a macroparametric effect obtains which corresponds to the system instantiated by the Palù variety. The mesoparameter corresponds to a system in which the movement to the non-finite verb form can only be found when v° is empty, i.e., in main declarative clauses. The fact that for a subgroup of speakers from Fierozzo and Roveda OV word order is accepted with modal verbs follows from a microparamter: the movement of the non-finite verb form to lowFocus° can only take place with non-finite verbs. Finally, the fact that OV is obligatory for nearly all groups is captured in terms of a nanoparamenter associated with negative constituents.

## Introduction

Minority heritage languages are one of the most complex contexts for linguistic description and analysis, since they represent a dialect of a language, which is spoken in isolation from other varieties and from the standard variety, typically in a bilingual/plurilingual environment by bilingual/plurilingual speakers (see papers in Putnam, 2011 for an overview of the situation of German-language islands). The presence of these two variables is problematic for linguistics because it indicates a high degree of variation, which is caused by the interplay of the typical sociolinguistic variables characterizing all dialectal variation, and the effects of contact/bilingualism.

Let us illustrate this complexity with the case of Mòcheno, a German minority heritage language spoken in three villages (Palù/Palai, Fierozzo/Vlarutz, Roveda/Oachlait) of the Fersina valley (Trentino, Italy) by around 600 people in a situation of triglossia (Trentino and Mòcheno are the lower varieties, bidialectalism, whereas regional Italian in the high variety. Standard German is a foreign L2 language^1^).

German and Romance traits co-occur in Mocheno, for example, in a sentence with a complex tense, the lexical verb can either appear at the end of the sentence as in 1a (building the brace construction, as in German, 1c), or after the auxiliary verb and before the object, as in Italian (1b/1d).


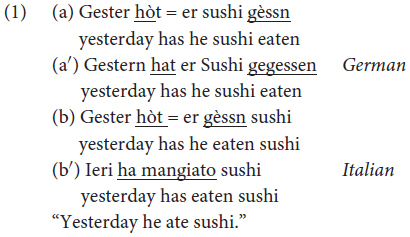


Since the kind of variation in (1) is an example of intra-speaker variation, that is an individual speaker accepts both word orders (Weiß, 2013. pp. 171–172) in Mòcheno, and since one of the two options coincides with the German order (1a–1c) and the other one coincides with the Italian/Trentino order (1b–1d), it is traditionally claimed that the observed variation is due to contact (Rowley, 2003). More specifically, the German option is assumed to be original, whereas the other is assumed to be acquired. This way of looking at data, which considers the orders coinciding with German to be original and those pattering with Italian to be an innovation, implies that Mòcheno has undergone a process of syntactic change due to contact with Romance languages (which is compatible with Kroch, 1989 double-base hypothesis).

Another way of looking at intra-speaker variation is to consider that the two orders are not a conservative and an innovative option, respectively, but coexist within a single grammar where they have specialized for the realization of different discourse functions, as Cognola (2013a,b, 2014) shows for OV/VO word orders, pro-drop (asymmetric pro-drop) and the Verb Second (V2) rule. According to these studies, which rely on fine-grained theory-informed descriptions of the facts, variation does not follow from the availability of two competing systems, but from rules internal to a single grammar. A key piece of evidence in favor of the single-grammar hypothesis is the fact that contexts/phenomena can be identified in which only a single word order is grammatical – which runs counter the predictions of the competing grammars hypothesis that predicts that two competing options should be always available (variation should be “rampant” in all investigated contexts Svenonius, 2000, p. 280).

Another source of variation that has received consistently less attention in the case of Mòcheno syntax is sociolinguistic variation, or the extent to which the observed mixed system is also subject to inter-speaker variation, particularly with regard to diatopic and diastratic variables. The notions of diatopic and diastratic variations, together with that of diaphasic variation, have been considered a key factor in sociolinguistic research since Coseriu (1980) work, and their interaction is assumed to shape the language’s dimensions of synchronic variation. The term diatopic variation refers to the synchronic variation found in a language due to the different geographical origins or distribution of speakers. Diatopic variation is found at all linguistic levels of analysis in British, American, and Irish English (macro-variation; see Milroy, 1987 for morphosyntactic phenomena), and within a single variety (micro-variation; for example, see the centralization of the first vowel in the diphthongs [aƱ] to [ƏƱ] and [aı] to [Əı], which Labov (1972) found to be a typical trait of the Up-island speech in his classic study of the variety spoken in Martha’s Vineyard.

Diastratic variation refers to synchronic variation within a language as a result of the different socio-cultural backgrounds of the speakers (according to age, sex, profession, and so forth). With reference to Labov (1972) study of the pronunciation of vowels in the Martha’s Vineyard variety, he found that centralization was subject to diastratic variation, since it was more frequent in the 31–45 age group and was slightly more frequent in those of Yankee descent and people making a living from fishing.

The last dimension of synchronic variation that has traditionally been claimed to play a role in sociolinguistic studies is that which is connected to different linguistic situations/contexts (formal, informal, and so on), and is called diaphasic variation. This dimension of synchronic variation does not appear to play any role at the syntactic level in Mòcheno, given that this language is spoken in a situation of bidialectalism with the local Trentino dialect, and is thus primarily a spoken variety used in informal situations. Due to the recent efforts in language planning that have led to the introduction of a writing system, Mòcheno has begun to be written in newspapers in the local press and to be used in formal contexts (administrative documents). According to Covi (2018) pilot study on diaphasic variation in Mòcheno, the use of the language in higher contexts does not appear to have an effect on its syntax, but only on lexical choice (German words are used more frequently in formal/administrative texts).

Given the unique situation of Mòcheno, in which both intra- and inter-speaker variation coexist, the aim of this paper is to provide a statistical analysis of the distribution of OV/VO word orders in different syntactic constructions.

With regard to the syntax of main clauses with a complex verb form and to embedded clauses Rowley (2003) claimed that Palù appeared to be a more conservative variety (OV maintained), whereas Fierozzo and Roveda are more innovative (VO is more frequent). Cognola (2013a, 2014) also pointed to diatopic variation between the two varieties with regard to the realization of the subject pronouns in main and embedded clauses (Palù is a symmetric language, whereas the varieties spoken in Fierozzo and Roveda are asymmetric; in other words, subject clitics are obligatory in main clauses and are ruled out in embedded clauses). With regard to the distribution of weak subject pronouns in *Scene setter – subj – V* configurations, these are extremely marginal in Roveda and possible in Fierozzo and Palù, but are subject to diastratic (age) variation in the latter variety (Cognola, 2013a, p. 92).

Moreover, (Cognola, 2013a, p. 155, Table 5.2) also shows for the distribution of subject-finite verb inversion in X-V-subj sentences that this construction is always accepted by the speakers of Palù (15/15) and by (20/30) speakers in Fierozzo and Roveda – which means that one third of the speakers of these varieties only accepts sentences without inversion.

These conclusions concerning Mòcheno syntax are derived from studies based on different empirical bases. The syntactic description in Rowley (2003) grammar relies on examples taken from written sources with no indication of information such as the age of the informant or the text used. The data in Cognola (2013a, 2014) studies conducted within the framework of Generative Grammar are more accurate since the data were collected through the scholar’s own fieldwork, but no real quantitative work has been done.

The aim of this paper is to contribute to our understanding of variation by providing a quantitative statistical analysis of a selection of its syntactic phenomena (see Van Craenenbroeck et al., 2018). The first issue we hope to shed new light on is the dimension of intra- and inter-speaker variation in Mòcheno. We will investigate the extent to which intra-speaker variation is found across all Mòcheno speakers, particularly in order to shed light on the ‘gray zone’ detected in Cognola (2013a, p. 155, Table 5.2) constituted by speakers only adhering to the Italian syntax. We will then address the main question of the role of contact in shaping Mòcheno syntax in an attempt to establish the extent to which variation is a direct result of contact.

The paper is organized as follows. In section “The Study,” we present the empirical basis of the study by discussing the phenomena addressed (the position of the finite verb in embedded clauses, in sentences with a modal verb, negative concord, the position of focused light/heavy objects in main clauses with a complex tense and scrambling), and the way in which our data were collected (own fieldwork). In section “A Statistical Analysis,” we present the results of the statistical analysis, which are discussed in section “Discussion of the Results.” Section “Conclusion” summarizes the results of this paper.

## The Study

In this section, we provide a description of the empirical part of the study.

In this paper, we discuss five different phenomena that have been investigated on two different occasions. The position of focused light/heavy objects in main clauses with a complex tense and scrambling were investigated in a previous survey that was conducted in two phases. In the first phase, single interviews with one informant from the village of Palù were conducted and a first description of the phenomena was derived. In the second phase, which involved 45 informants, the hypothesis constructed based on the data obtained from the single (first) informant were tested on a wider scale (Cognola, 2013a). The data collected in this survey had never been investigated from a statistical perspective; thus, we decided to include them in this paper in order to provide a more solid empirical basis for our statistics. The other three phenomena – the position of the finite verb in embedded clauses and in sentences with a modal verb, and negative concord – had never been investigated from a theory-informed perspective, and were studied in a novel study conducted in 2018, the results of which are presented in this paper for the first time. As discussed in more detail below, the data collection for this last study was carried out in two phases. In the first phase, three informants, one for each language variety, were asked for translations and grammaticality judgments regarding a variety of sentences. Based on the data collected in this phase, a questionnaire was designed and tested with 55 informants. Table 1 summarizes the studies on which the present paper is based.

**TABLE 1:** Overview of the studies discussed in the paper.

	**Phenomena tested**	**Pilot study (Phase 1)**	**Questionnaire study (Phase 2)**	**Judgment scale**
Study 1	OV/VO; scrambling	1 informant from Palù	45 informants whole valley	1 (fully ungrammatical) -2-3 (fully grammatical)
Study 2	OV/VO in embedded clauses; OV/VO in sentences with a modal verb; negative concord	3 informants, 1 for each variety	55 informants whole valley	1 (fully ungrammatical)-2-3-4-5 (fully ungrammatical)

All informants involved in the two studies were born in the Fersina valley, have always lived there, and share the same sociolinguistic situation described in section “Introduction.”

### Investigated Phenomena

#### Position of the Finite Verb in Embedded Clauses

According to the literature on Mòcheno (Rowley, 2003, p. 288), the finite verb can appear in one of two positions in embedded clauses, either in the sentence-final position (2a) or in the same position as in main clauses (2b). In the latter case, the so-called *Satzklammer* (brace construction, see Höhle, 1986 for this terminology proposed within the topological field model used to describe the German clause) can arise (2c), as in main clauses (all examples are taken from Rowley, 2003, p. 288).


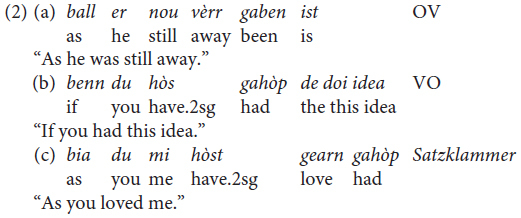


No detailed study on the distribution of the three word orders in all types of embedded clauses is given in Rowley (2003) grammar, but the following information is provided:

(3) *Mòcheno shares with German the fact that the position of the finite verb differs in main and embedded clauses. However, the Italian model characterized by the same position of the finite verb in main and embedded clauses is becoming established (*Rowley, 2003, p. 289, translation by F.C.).

(4) *As an effect of the contact with Italian the finite verb can appear in the same position in main and embedded clauses* (Rowley, 2003, p. 291, translation by F.C.).

Rowley (2003) claim relies on the fact that the OV syntax observable in Mòcheno embedded clauses corresponds to the word order found in German and, conversely, the VO word order corresponds with the linear word order of Italian, as shown in (5).


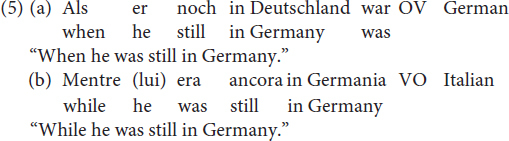


Moreover, from the grammar, we learn that:

(6) *Originally, and mostly still in Palù, the finite verb must appear in the sentence-final position in embedded clauses* (Rowley, 2003, p. 289, translation by F.C.).

What we learn from the grammar is that the strict OV word order is assumed to be a German option, whereas the VO option corresponds to the word order found in Italian. The latter is presumably getting more space due to contact with this language as an effect of bilingualism. With regard to diatopic variation, the grammar claims that the variety spoken in Palù appears to feature OV more frequently, whereas the dialects of Fierozzo and Roveda tend to use VO more often.

#### OV/VO Word Orders in Main Clauses Featuring a Modal Verb

In the literature (Rowley, 2003, p. 279), it is documented that two word orders are possible in main clauses featuring an auxiliary verb and a past participle or a with modal verb and an infinite verbs: either VO word order (8a) or the *Satzklammer* (8b,c, all examples are taken from Rowley, 2003, p. 279).


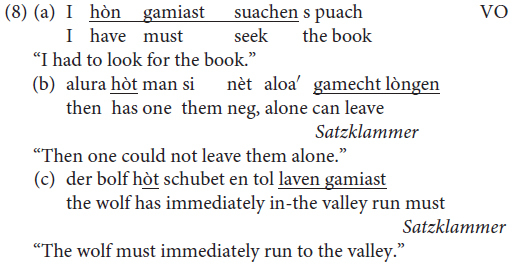


As in the case of embedded clauses discussed in section “OV/VO Word Orders in Main Clauses Featuring a Modal Verb,” the two options available in Mòcheno correspond to the word orders found in German and in Italian, as shown in (9).


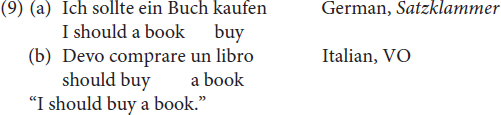


With regard to the sociolinguistic distribution of the two options in (8), we learn from the grammar that the VO option is mainly used in Roveda and Fierozzo, whereas the *Satzklammer* is also found in Palù.

There is no mention of the discourse-information status of the arguments appearing in sentences with modal verbs in the grammar. According to Cognola (2013a), Cognola and Bidese (2013), and Cognola and Moroni (2018) studies of the distribution of arguments with complex verb forms (with *to be* and *to have* auxiliaries), the position before the non-finite verb in main clauses can only host focused constituents, whereas topics are banned. This correlates with the fact that the *Satzklammer* is ruled out in wh-interrogative clauses – a fact that Cognola (2013a) attributes to the fact that (i) only foci can precede the non-finite verb forms that are hosted in a LowFocusP of the vP periphery (Belletti, 2004; Poletto, 2006); and (ii) when a focus or a wh-element is extracted from the lower phase, it first saturates the LowFocusP due to the cyclicity of movement (Chomsky, 2001). With regard to the distribution of OV/VO word orders, Cognola and Bidese (2013) data indicate that the presence of intra-speaker variation is more frequent in this variety, but it is not excluded from other varieties.

Whether the correlation between the ability of a constituent to appear in the *Satzklammer* and its information status also holds for sentences featuring a modal verb was not discussed by Cognola (2013b) or Cognola and Moroni (2018).

#### Negation

In Mòcheno, sentential negation is realized through the negative particle *nèt* “not” (examples are taken from Rowley, 2003, p. 261).


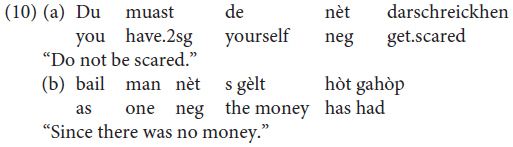


Constituent negation is realized through the negative pronoun *khoa* (data from our study, sentences judged as being perfectly grammatical by all three informants, one for each variety).


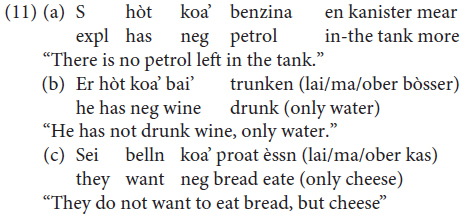


However, *nèt* can also be used to negate a constituent. In this case, the negated XP gets a focus reading and the presence of a correction is required (data from our study: sentences judged as being perfectly grammatical by all three informants):


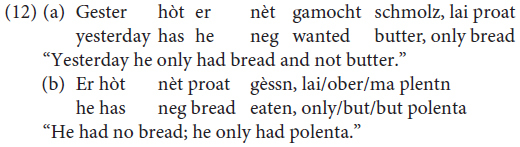


(Rowley, 2003, p. 263) claims that *negative concord* is possible, but not obligatory, in Mòcheno:


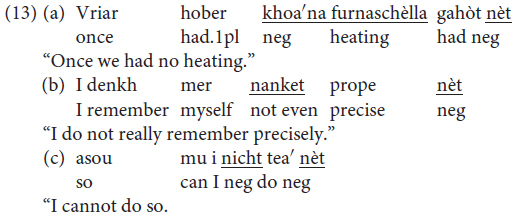


The possibility of negative concord in Mòcheno is reminiscent of the phenomenon we can observe in Italian – a language characterized by non-strict negative concord (Giannakidou, 2000).


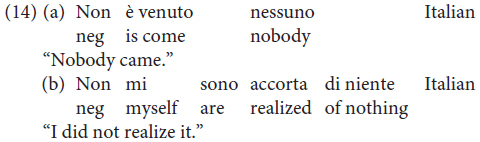


In standard German, by contrast, negative concord is not possible: the presence of a negative XP blocks the insertion of other negative constituents.


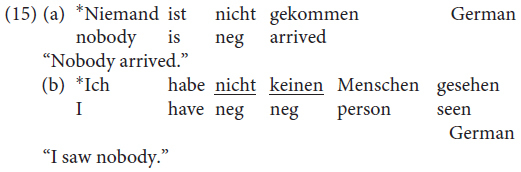


The data regarding negation thus replicate what appears to be a recurring pattern of variation in Mòcheno syntax: two options appear to be available, and they correspond to the linear word order of present-day German and present-day Italian. Note, however, that negative concord is possible in a reduced number of West Germanic languages, amongst which are Bavarian dialects (see Weiß, 2002; Biberauer and Zeijlstra, 2012 with reference to Afrikaans).

#### Summary

The position of the finite verb in embedded clauses, the position of the finite verb in sentences featuring a modal verb, and the syntax of negation represent three privileged phenomena whereby syntactic variation can be investigated. According to the literature, Mòcheno exhibits at least two of these options in these phenomena, which correspond to the word orders found in present-day German and present-day Italian. Therefore, these phenomena are an ideal testing ground for the study of the interplay between syntactic variation and contact. In order to do so, in our study, we addressed a series of facts that have been neglected in the literature. More specifically, we wanted to determine the extent to which the syntactic variation documented in the literature and discussed above in sections “Position of the Finite Verb in Embedded Clauses,” “OV/VO Word Orders in Main Clauses Featuring a Modal Verb,” and “Negation” can be considered an example of intra-speaker or inter-speaker variation.

### Methodology

In order to address the research questions in section “Summary,” we carried out fieldwork in two phases. In the first phase, we worked with three carefully selected, middle-aged informants, one for each language variety.

In the first study, the informants were asked to provide grammaticality judgments using a five-point scale (1 = completely ungrammatical; 5 = completely grammatical) for 233 sentences featuring negative elements. In this detailed questionnaire, we tested:

(i) the position of all types (pronouns, bare quantifiers, quantifier phrases, negative adverbs and modal particles) of negative constituents with regard to the finite and non-finite verb forms in a variety of sentences (featuring modal verbs, other complex tenses, simple tenses);
(ii) their co-occurrence within the same clause;
(iii) the negative adverbs used to answer positive and negative questions.

We found that, for all informants, double negation was always ruled out, irrespective of the XPs combined (pronouns, adverbs, and negative particles) and their relative word order. Moreover, all negative subjects and objects (pronouns, PPs, bare quantifiers, and quantifier phrases) must appear before the non-finite verb form within the *Satzklammer* and not after it (VO).

Using the same informants, we tested the distribution of subjects and objects in main declarative (226) and interrogative (89) clauses featuring a modal verb. We tested all the modal verbs (*belln* “want”; *derven* “be allowed”; *meing* “can”; *khennen* “can, be able”; *schellten* “must”; *miasn* “must”) and the periphrastic form *tea’*+ infinitive. What we found was that the presence of all modal verbs appeared to favor the position of a DP subject or an XP object within the *Satzklammer* in Palù, irrespective of its information status and of the type of sentence in which it appeared. This is a highly relevant result, since Cognola (2013b) and Cognola and Moroni (2018) indicated that, for the Palù variety, the preverbal position within the *Satzklammer* was not permitted for non-focused XPs with complex verb forms featuring the auxiliary verb *to be* and *to have* in main clauses, and in all interrogative clauses. The situation appears to be less clear for Roveda, possibly because we do not have as detailed a study of the distribution of OV/VO word orders in this variety as we have for Palù.

With two informants, one from Palù and one from Fierozzo, we then studied the distribution of OV/VO word orders in embedded clauses via a questionnaire featuring 30 types of embedded clauses for which three word orders (OV, VO, and *Satzklammer*) were presented to informants, who were asked to provide grammaticality judgments (using a five-point scale with 1 = completely ungrammatical and 5 = completely grammatical). What we found was that informants diverged in their judgments of nearly all sentences; in other words, when an order was judged to be perfect by one informant, it was rated 1 by the other (the same judgments occurred in 17/90 sentences, 18%).

Based on these preliminary results, we designed a questionnaire consisting of 57 sentences which is available in the Supplementary Material associated with this paper. When selecting the sentences to test on a wider scale, we were guided by the necessity of having a relatively short questionnaire that could be completed in a single work session. In light of the specific geographical configuration of the Fersina Valley (scattered farms, mostly isolated and far from each other), it would have been unrealistic to plan a large-scale study in which more than a single questionnaire could be tested, since the risk would be that the data collection would not be completed. However, the questionnaire also needed to be effective in testing precisely those key contexts that were crucial with regard to the phenomenon.

With regard to negation, we decided to test only:

(i) the position of the negative bare quantifier and the quantifier phrase with regard to the non-finite verb (two sentences);
(ii) a selection of cases of negative concord (all of which were judged as being ungrammatical by our informants, whereas the sentences featuring a single negation were scored perfect by being assigned a 4); and
(iii) the distribution of answering adverbs across different contexts (not considered here).

Only five sentences were considered in this study: two considered the position of the negative quantifier phrase with regard to the non-finite verb form, and three considered negative concord. In (16) is an example of each type of sentence in the form in which it was presented to the informants via the questionnaire.^2^


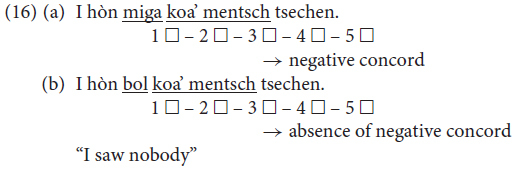


Non userei nessuna di queste frasi e direi invece

I would not use any of the proposed sentences and I would say instead…….


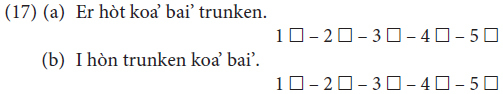


Non userei nessuna di queste frasi e direi invece

I would not use any of the proposed sentences and I would say instead……

For each embedded clause, informants were presented with three alternatives featuring OV, VO syntax and the *Satzklammer*, and were asked to judge the grammaticality of the options proposed using a discrete scale ranging from 1 (completely ungrammatical) to 5 (completely grammatical).^3^ See Table 2 for a complete list of embedded clauses tested.

**TABLE 2:** Types of embedded clauses tested.

**Clause type**	**Introductory element**	
Embedded interrogative	*Babai* (why)	2
	*Bos* (what), direct obj	1
	*Ber* (who), subject	1
	*En bem* (whom), indirect object	1
	*Biavle lait* (how many people), subj	1
	*S beil puach* (which book), direct object	1
	*Benn* (when)	1
	*Benn* (if)	1
	*Abia* (how)	1
	*Bos ver +NP* (what kind of) (subj)	1
	*Bo* (where)	1
Completive clauses	*As*	5
Temporal clauses	*Derbail* (while)	1
	*Benn* (when)	1
	*Derno as* (after that)	1
	*An iats vort as* (that time)	1
Modal	*Abia as*	1
Causal	Babai	4
Exclusive	*A’ne as* (without that)	1
	*Aus (*without)	1
Conditional	*Benn* (if)	2
Concessive	*Benn aa* (although)	1
Consecutive	*as* (so that)	1
Comparative	*abia benn* (as if)	1
Relative clause	*as* (that)	4

In (18) we give an example of a task for an embedded clause.


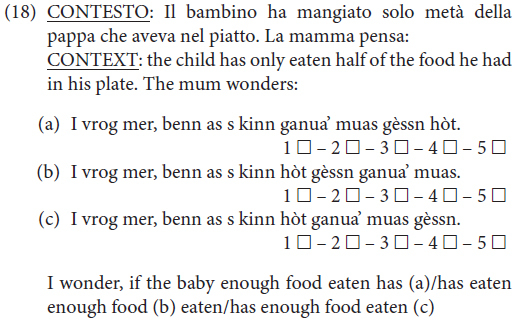


Non userei nessuna di queste frasi e direi invece:

I would not use any of the proposed sentences and I would say instead……

The last phenomenon, namely the distribution of arguments with regard to the non-finite verb form in the *Satzklammer* with modal verbs, was investigated using 13 sentences.^4^ We considered (i) sentences involving a focus reading of the constituent preceding the non-finite verb form (five sentences), (ii) wh-interrogative clauses (three sentences), and sentences in which no context was given (five sentences).

We judged this number of sentences to be sufficient to test whether modal verbs favored the position of arguments within the *Satzklammer* on a wider scale irrespective of the information structure and clause type, based on the information provided by the consulted informant from Palù. The modal verbs we considered were *belln* “want”; *derven* “be allowed”; *meing* “can,” *khennen* “can, be able” and *schellten* “must.” We also considered the periphrastic form *tea’*+ infinitive. In (19), an example of how sentences were presented to the informants is shown.


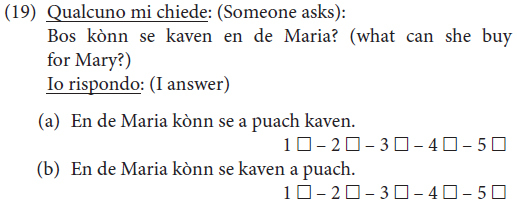


To Mary she can a book buy/To Mary can she buy a book

Non userei nessuna di queste frasi e direi invece:

I would not use any of the proposed sentences and I would say instead……..

More than one alternative was tested for all three phenomena and for all the sentences tested. For the embedded clauses, we tested three possible word orders (the ‘German’ OV, the ‘Italian’ VO and the ‘mixed’ *Satzklammer*); for modal verbs, we tested the ‘German’ *Satzklammer*, the ‘Italian’ VO and the fronting of the object. Two options were tested for negation, which could be considered ‘German’ in two of the five sentences (that is, three sentences featured negative concord).

In Table 3, we summarize the number of sentences for each phenomenon tested in the questionnaire.

**TABLE 3:** Phenomena tested.

**F number**	**Phenomenon**	**Number of sentences**	**Alternatives tested**	**Alternatives considered^1^**
F1	Embedded clauses	37	3	2
F2	Negation	5	2	2
F3	Modal verbs	13	3	2

*^1^We excluded the results of the third column because we wanted to tested how much Mòcheno grammar sticks to German or Italian syntax. Since the *Satzklammer* in embedded clauses and the fronting with modal verbs represent a sort of “mixed” options, we did not consider them in our study.*

### Population

We administered the questionnaire to a total of 55 informants^5^ who could express their acceptance/rejection of all the options proposed on a discrete scale ranging from 1 (complete refusal) to 5 (complete acceptance). The questionnaire was handed to informants by assistants of the Institute for the Promotion of the Mòcheno Language and Culture. The informants received detailed instructions and detailed explanations regarding what they were required to do in terms of the questionnaire – they completed the questionnaires on their own at home. Then questionnaires were returned to the assistants from the Institute for the Promotion of the Mòcheno Language and Culture. The results of each questionnaire were copied into an Excel file by Evelina Molinari.

All informants were selected carefully by paying special attention to the specific sociolinguistic situation of Mòcheno (see Cognola, 2013a). They were all considered excellent speakers in the community and were completely reliable (see Dorian, 1989).

The informants were selected by considering diatopic and diastratic (age) variations (see Coseriu, 1980; Cognola, 2013a). As summarized in Table 4, we attempted to obtain an appropriate number of informants from the three villages (Palù, Fierozzo, and Roveda); we also attempted to ensure a good balance across age groups (younger speakers: younger than 30; middle-aged speakers: 30–60; elderly speakers: older than 60).

**TABLE 4:** Number of informants involved in the questionnaire study.

**Village**	**Number of informants**	**Young**	**Middle-aged**	**Elderly**
Palù	16	6	5	5
Fierozzo	20	5	8	7
Roveda	16	6	5	5

The villages in the Fersina valley are composed of several scattered farms – a situation that might lead to micro-diatopic syntactic variation within a single variety (see Cognola, 2013a, Chap. 1 and p. 131 for an example of this taken from the variety of Palù). Therefore, in the selection of informants, we attempted to include most of the farms.

We also attempted to ensure a good gender balance (even though Cognola, 2013a has shown that gender does not appear to be a relevant factor for sociolinguistic variation in Mòcheno) in all the groups, but this was not possible due to the fact that male informants were considerably less forthcoming than were female informants. Therefore, we had to exclude some of the questionnaires that were completed by men because not all the questions were answered appropriately.

### More About OV/VO Alternations

In this paper, we also considered data that were collected in a previous survey but which had never been analyzed statistically (data collected for Cognola, 2013a). In that survey, the distribution of OV-VO orders involving a focused light or heavy object in sentences involving a complex tense (non-modal) in main declarative clauses was considered. Contexts involving a focused subject were tested because, according to Cognola (2013a) and Cognola and Moroni (2018), the preverbal position in main clauses can only host focused constituents, whereas topics are banned.

In the survey, informants were asked to translate four sentences that constituted an answer to an object main interrogative clause, and to evaluate the two alternatives that they did not produce.


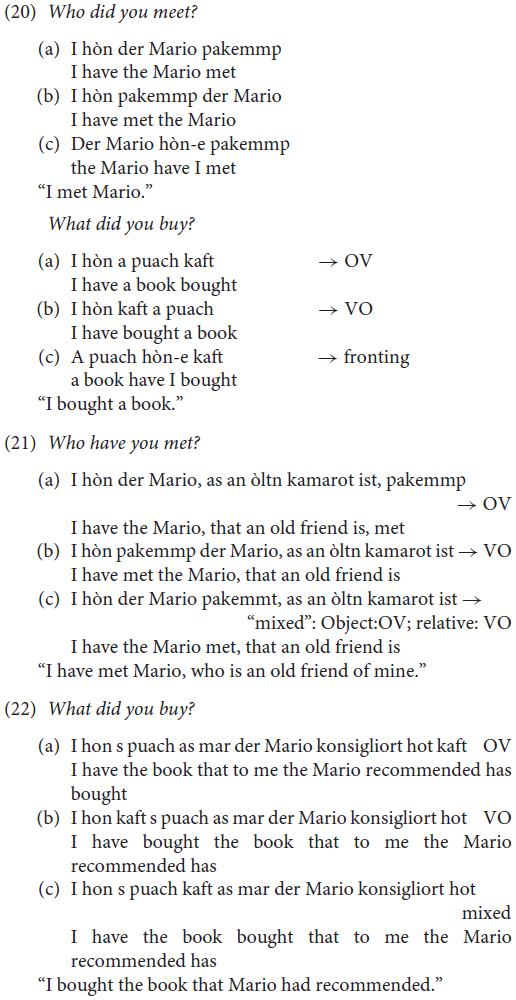


The second phenomenon we considered was scrambling above sentential adverbs (see Cognola, 2017). In the previous survey (Cognola, 2013a), three sentences containing this type of scrambling were tested: one involving the subject (23a), one involving the object (23b) and one involving both (23c), along with two sentences in which the arguments appeared below sentential adverbs (unmarked word order in Mòcheno; see Cognola, 2017).


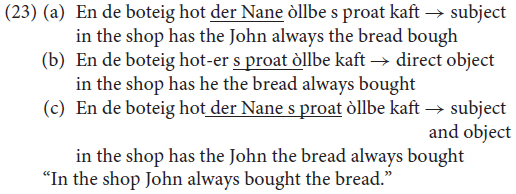


The survey involved 45 informants (plus three controls, one per village) who were selected according to the sociolinguistic criteria. The phenomena were included in a syntactic questionnaire that was presented orally to informants by Federica Cognola, who interviewed each of them in single 1-h sessions. The data were then transcribed by Federica Cognola into a separate file for each informant.

For each informant, and for each sentence, we assigned a value of +1 to the proposed order (translation), 0 to the accepted alternatives, and −1 to the rejected forms.

## A Statistical Analysis

### Presentation of the Results

In this section, we detail the statistical approach we used to describe the data.

**FIGURE 1:**
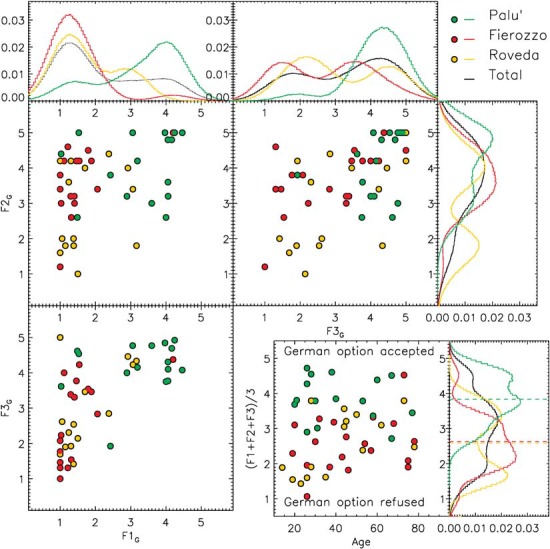
Distribution of the average evaluations of the ‘German’ option, for three different phenomena (F1 = syntax of embedded clauses, F2 = syntax of negation and F3 = syntax of main clauses with a modal verb). The histograms show the normalized, smoothed distributions of the real data along F1, F2, and F3. For the smoothing, we used a Gaussian filter defined by σ = 0.5. In the bottom-right panel, we show the distribution of the average evaluation given by each informant for the three phenomena presented according to the ages of the informants. Each data point corresponds to a single informant. The average values obtained in Palù, Fierozzo, and Roveda are reported consistently in the bottom-right histogram using dashed green, red, and yellow lines, respectively. The bottom-right histogram represents the sum of the smoothed distributions computed for the three phenomena considered individually.

**FIGURE 2:**
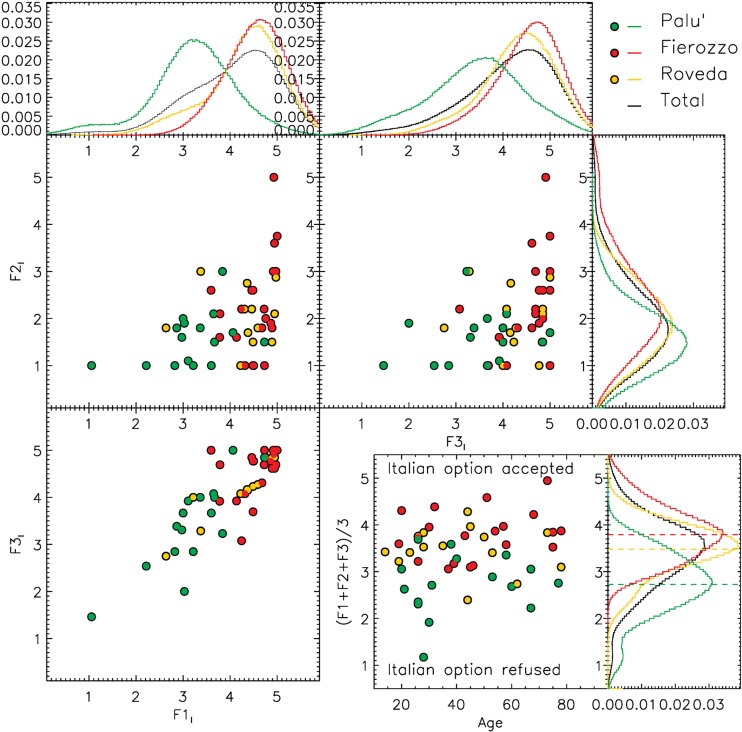
Distribution of the average evaluations for the ‘Italian’ option for three different phenomena (F1 = syntax of embedded clauses, F2 = syntax of negation, and F3 = syntax of main clauses with a modal verb). The histograms show the normalized, smoothed distributions of the real data along F1, F2, and F3. For the smoothing, we used a Gaussian filter defined by σ = 0.5. In the bottom-right panel, we show the distribution of the average evaluation given by each informant for the three phenomena combined, presented according to the ages of the informants. Each data point corresponds to a single informant. The average values obtained in Palù, Fierozzo, and Roveda are reported consistently in the bottom-right histogram using dashed green, red, and yellow lines, respectively. The bottom-right histogram represents the sum of the smoothed distributions computed for the three phenomena considered individually.

**FIGURE 3:**
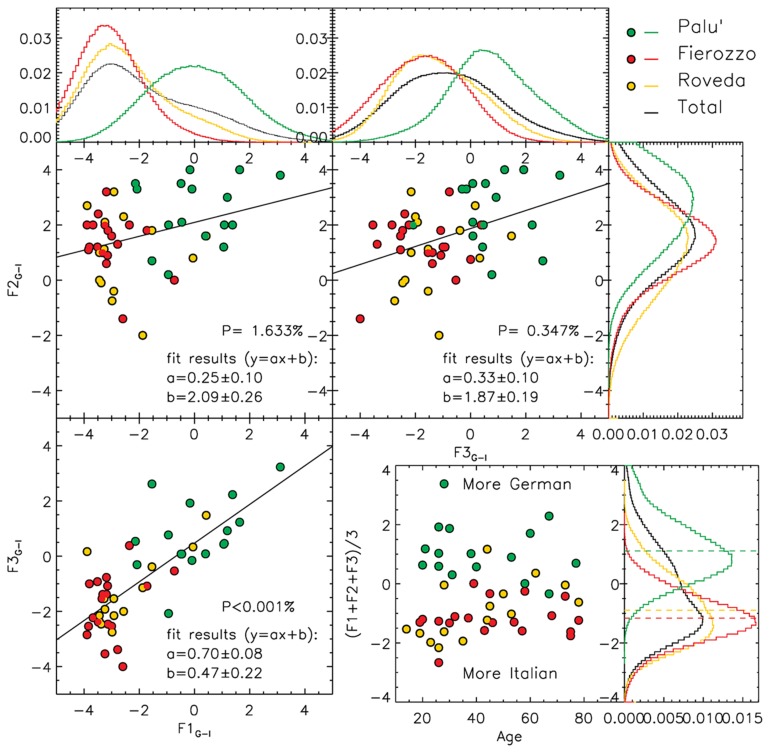
Distribution of the values obtained by subtracting the results measured for the ‘Italian’ option from those obtained for the ‘German’ option. Three phenomena (F1 = syntax of embedded clauses, F2 = syntax of negation, and F3 = syntax of main clauses with a modal verb) are considered (the two upper and the bottom-left panels). The histograms show the normalized, smoothed distributions of the data along F1, F2, and F3. For the smoothing, we used a Gaussian filter defined by σ = 1.0. In each plot, we computed the linear fit for the data, finding significant correlations (*p* < 2%) in all the three combinations. In the bottom-right panel, we show the distribution of the average German-Italian evaluation given by each informant for the three phenomena combined presented according to the ages of the informants. Each data point corresponds to a single informant. The average values obtained in Palù, Fierozzo, and Roveda are reported consistently in the bottom-right histogram using dashed green, red, and yellow lines, respectively. The bottom-right histogram represents the sum, element by element, of the smoothed distributions computed for the three phenomena considered individually.

**FIGURE 4:**
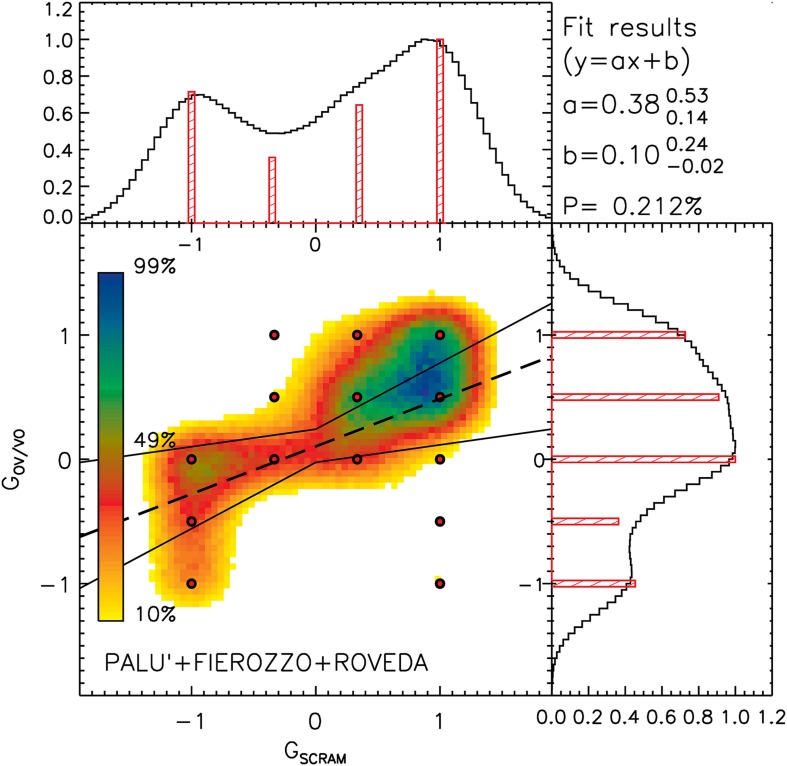
Distribution of the normalized values for G_SCRAM_ and G_OV/VO_ for the all the informants (red circles). The minimum and maximum values shown in the plot (–1 and +1) correspond to the minimum and maximum values of the non-normalized G_SCRAM_ and G_OV/VO_ found in the survey. The normalized distribution of these data along the x and y axes are shown in the horizontal and vertical histograms (red columns). After smoothing the distribution of the real data (σ_x_ and σ_y_ correspond to the average distance between the bins of the discretized distributions along x and y), we obtained the distribution shown in the plot using the color scale and the horizontal and vertical histograms (black curve). The linear fit and the 3σ associated uncertainties are shown using one dashed and two continuous black lines.

Most of the analyses are based on a descriptive statistical approach. In this sense, Figures 1–4 show the distributions of the average answers given by the informants under different circumstances (phenomena considered, diatopic and diastratic variables). The data shown in Figures 1–4 are available in the Tables in the Supplementary Materials associates with this paper. To improve the intelligibility of all the plots, besides showing the actual position of the original data points (each of which corresponds to a single informant), we smoothed the original distributions of the data by applying a Gaussian filter. This method allowed us to mitigate the effects generated by the discretized scales that the informants were allowed to use during the surveys. The smoothing scale used was not arbitrary, since it was always calibrated on the level of discretization of the original data (defined as the inverse of the number of possible average answers that an informant could provide). The effects of the discretization are particularly visible in Figure 4, in which the original data points overlap in the nodes of the grid of the possible values accessible in that particular survey. Such a data distribution strongly limited the intelligibility of the original data. For this particular case, we show the smoothed distribution not only in a separate histogram (as we do in all other cases), but also as a 2D density map coded using a color scale.

Besides the statistical description of the data, in Figures 3, 4, we tested the null hypothesis using a *p*-value test, according to which data are distributed randomly. We always obtained low values for *p*, indicating that the null hypothesis needed to be rejected. Following this result, we describe the dependence of the different phenomena by using a linear fit (the associated uncertainties are also reported in both Figures 3, 4, and are shown in Figure 4).

#### Embedded Clauses, OV/VO With Modals and Negation

We studied the distribution of the judgments given by informants for the German and the Italian options in the three phenomena.

In Figure 1 (upper and bottom-left panels), we show all the x versus y combinations of the F1, F2, and F3 phenomena for the German option (F1_G_, F2_G_, F3_G_). The data points are color-coded to represent the average answers given by each of the informants from Palù (green), Fierozzo (red), and Roveda (yellow). The same color coding has been retained to represent the distributions of the same data points in the corresponding histograms (the distribution of the entire sample is represented in black). In order to provide a clearer view of the results of the survey, the distribution of the original average answers (represented in the plots) in the histograms have been smoothed using a Gaussian filter with σ = 0.5, which is half of the step’s width separating two possible answers (the informants could answer 3 or 4, for example, but not 3.2 or 3.75).

The bottom-right panel in Figure 1 represents the average answers F_G_ given by each of the informants, computed for the three phenomena for the German option (G) as a function of their age: F_G_ = (F1_G_+F2_G_+F3_G_)/3.

We report the smoothed distribution of the actual data in the corresponding histogram.

The first important observation that we obtained from the plots in Figure 1 was that informants from Fierozzo and Palù clearly showed opposing attitudes with regard to the ‘German’ option. While the ‘German’ option was generally accepted by the informants from Palù (the distribution over F1_G_, F2_G_, and F3_G_ always peaked between ∼4 and ∼5), the same option tended to be refused (F1_G_) or was not accepted unanimously (F2_G_ and F3_G_) by the informants from Fierozzo. This resulted in F_G_ = 2.63 ± 0.10 for Fierozzo and F_G_ = 3.83 ± 0.09 for Palù. The average attitude in Roveda (F_G_ = 2.58 ± 0.13) was in agreement with that measured in Fierozzo, but the data distribution depicts a more controversial scenario. While the answers given by informants from Fierozzo and Palù tend to be coherent within each age group (with the possible exception of F3_G_ for Fierozzo), informants from Roveda tended to divide themselves into subgroups (this was particularly clear for F2_G_ and F3_G_), although this was not a straightforward trend and closely reproduced the attitude of informants from Fierozzo on occasion (as in F1_G_ and F3_G_). This situation is represented well by the measure of the standard deviations (that is, the spread of the data around ≪*cps*:*it* > *Fi* < /*cps*:*it*≫), which correspond to 0.80, 0.63 and 0.88 for Fierozzo, Palù, and Roveda, respectively.

The second relevant piece of information shown in Figure 1 (bottom-right panel, where the variable age is considered) is that, for the informants from Palù and Fierozzo, <F_G_> was not correlated significantly with age. In other words, the average acceptance or rejection of the German option was mainly due to geographical reasons (residence), as explained above, and was not based on the age of the informants.

Again, the case of Roveda is in contrast with the previous statement: the bottom-right panel in Figure 1 shows that informants older than 40 behaved differently from younger informants. In particular, with the exception of one person, all the informants from Roveda who were younger than 40 behaved consistently with the behavior we observed for the case of Fierozzo (<F_G_> ∼1.5), clearly rejecting the German option. Instead, the answers of the informants from Roveda who were older than 40 were more consistent with an average weak acceptance of the German option (<F_G_> ∼3). Therefore, this sub-group positioned itself halfway between Palù and Fierozzo.

In the plots in Figure 2, we show the judgments of the informants regarding the Italian option for the three phenomena (F1_I_, F2_I_, and F3_I_). In all three groups (Fierozzo, Roveda, Palù), the Italian word order was commonly accepted in the F1_I_ and F3_I_ cases and rejected in F2_I_. However, we also observed significant differences between the groups from Fierozzo and Palù, with the informants from Palù being relatively less enthusiastic about this option, with < F1_I_ > ∼3 and < F3_I_ > ∼3.5, whereas acceptance was more evident in the group from Fierozzo, with < F1_I_ > ∼ < F3_I_ > ∼ 4.5. The distribution of the data points corresponding to the group of informants from Roveda can be assimilated with those observed for the group from Fierozzo, without significant differences. As shown in the bottom-right panel of Figure 2, summing the results of the three phenomena, we obtain < F_I_ > = 3.790.07, 2.73 ± 0.09 and 3.48 ± 0.07, with standard deviations of 0.52, 0.65 and 0.48 for Fierozzo, Palù, and Roveda, respectively.

Finally, we subtracted the results obtained for the Italian option from those obtained for the German option for each phenomenon in order to create a scale on which a more German attitude corresponded to higher (positive) values, and a more Italian attitude corresponded to lower (negative) values. The results are shown in Figure 3. As in the previous figures, a double distribution emerged, with informants from Palù showing a more ‘German’ (or less ‘Italian’) attitude toward the three phenomena, and informants from Fierozzo and Roveda showing a more ‘Italian’ (or less ‘German’) attitude toward the same phenomena. This behavior was confirmed when averaging the results of F1_G–I_, F2_G–I_, and F3_G–I_ (bottom-right panel of Figure 3): the distribution of F_G–I_ peaked at positive values (<F_G–I_ > = 1.11 ± 0.10) for Palù, and at negative values (<F_G–I_ > = −1.16 ± 0.06 and −0.90 ± 0.1) for Fierozzo and Roveda.

We computed the linear fit for the distributions of data in the F1_G–I_, F2_G–I_, and F3_G–I_ space, and found a significant, direct correlation between the answers given in all these three cases with *p*-values that were always smaller than 2%. This result indicates that, for each informant, a more ‘German’ (or more ‘Italian’) attitude toward one of the three phenomena was usually associated with a similar attitude toward all the other phenomena.

Moreover, combining the results for the German and Italian options, we still did not observe a significant correlation with the age of the informants from Fierozzo and Palù. Instead, similarly to what we observed for the case of the German option, we observed different behavior on the informants from Roveda who were younger and older than 40 years of age. While young informants from Roveda show similar results to those from Fierozzo, the older ones represented a halfway point between the results observed in Fierozzo and in Palù.

#### Other OV/VO Alternations

Let us now provide a statistical analysis of the phenomena studied in the survey discussed in section “More About OV/VO Alternations.” Recall that, in the survey, the informants were asked to provide grammaticality judgments regarding the position of objects (VO, *Satzklammer*) in main declarative clauses involving a light (20) or a heavy focused object (21, 22), and on scrambling above sentential adverbs involving the subject, the object and both (23).

In this survey, speakers were not asked to rate the grammaticality of a sentence using a 1-to-5 point scale, but using a 1-to-3 point scale. Therefore, we need to associate the collected data with a numerical scale in order to perform a statistical analysis similar to that performed in section “Embedded Clauses, OV/VO With Modals and Negation.”

The survey involved 45 informants (plus 3 controls, one per village); for each informant, and for each sentence, we assigned a value of +1 to the proposed order (translation), 0 to the accepted alternatives, and −1 to the rejected forms.

With regard to the OV-VO order phenomenon, we quantified the informants’ judgments of the OV or the mixed order (G_OV/VO_) by summing the results obtained for the OV and the mixed forms (VO was not considered). Only one of the possible translations was ‘proposed’ by the informants (+1 assigned) for each of the two sentences, while the others were only considered to be ‘acceptable’ alternatives (0 assigned). For this reason, the maximum possible value of G_OV/VO_ corresponds to +2. By contrast, both the OV and the mixed order could be rejected simultaneously (−1 assigned) in both sentences if the VO order, which we are not considering, were the proposed form. Therefore, the minimum possible value for G_OV/VO_ corresponds to −4. In the survey, the actual minimum and maximum values for G_OV/VO_ obtained were −4 and 0, corresponding to a complete rejection and to an average acceptance of the OV and the mixed phenomenon, respectively. To avoid confusion, we finally re-normalized the maximum and minimum values obtained in the survey as −1 and +1, where +1 corresponds to the highest value of G_OV/VO_ obtained (0) and −1 corresponds to the lowest (−4).

Similarly to what we did for the OV/VO order, we tested the grammaticality of five possible alternatives (one of which was proposed by the informants) for the scrambling phenomenon. For this analysis, we considered only the three alternatives in (3) above in which the object appears before sentential adverbs. We computed G_SCRAM_ as the sum of the value obtained from the three alternatives. Since all three alternatives considered could be rejected at the same time, but only one of them could be proposed, the minimum and maximum values for G_SCRAM_ corresponded to −3 and +1. In the survey, the effective minimum and maximum values reached were −3 and 0. With regard to the OV/VO order, we re-normalized these values to −1 and +1, corresponding to a complete rejection and to an average acceptance of the scrambling phenomenon.

Given the nature of the survey, the possible values for G_OV/VO_ and G_SCRAM_ could only assume a few discrete values, corresponding to a grid of data points in a 2D space, in which x corresponds to G_SCRAM_ and y to G_OV/VO_. In Figure 4, we show the distribution of the data points in the 2D space described by using red circles. Since the values for G_OV/VO_ and G_SCRAM_ can only assume a few possible values between −1 and +1, many data points are superimposed upon the same x,y locations in the 2D space. The horizontal and vertical histograms in the same figure show the fractional distribution of the data along each x and y position (red columns). It is possible to see that _SCRAM_ axes (upper histogram), our data show a double peak in the distribution (one peak located at +1 and the other at −1) along the GSCRAM axes (upper histogram), indicating the existence of the two main differing attitudes of the informants with regard to the scrambling phenomenon, namely a complete rejection and an average acceptance of the scrambling phenomenon.

We computed the linear fit (y = ax+b) for the data, and obtained a positive correlation with a = 0.38 (3σ uncertainties: a_max_ = +0.53 and a_min_ = +0.14) and b = 0.10 (3σ uncertainties: b_max_ = +0.24 and b_min_ = −0.02). The linear fit and the associated 3σ uncertainties are shown in the plot using a dashed line and two continuous lines, respectively. The *p*-value associated with this relationship is *p* = 0.2%. The positive relationship that we found indicates that the use/acceptance of the scrambling phenomenon and of the OV order were related to each other: people accepting/preferring OV were usually also prone to accepting/preferring scrambling.

The uncertainties associated with the linear fit were computed as follows: for all 38 data points, each of which corresponded to one of the informants, we simulated 100.000 data points (3.8 million data points in total) distributed as the original data but with a different random quantity added. The distribution of the random quantity that we added was normal, with σ_x_ and σ_y_ selected to coincide with the average distance between the bins of the discretized distributions along x (G_SCRAM_) and y (G_OV/VO_). This operation should be equivalent to having more informants available for the interview and asking them to use a continuous scale of values ranging from −1, to 1, and not just the actual choices of −1, 0, and +1). This equivalence is valid only when assuming that an arbitrary, large number of informants would behave in exactly the same manner as the group that was considered in our survey (in proportion), and that the informants would make their choices in a normally distributed way along the discretized values from which they could choose. In our simulation, for 100.000 cycles, we simulated a new combination of 38 items of randomized data in the way described above, computing the ‘a’ and ‘b’ parameters of the linear fit each time. Finally, we assumed the 0.15 and 99.85% percentiles of the ‘a’ and ‘b’ parameter distributions as the 3σ uncertainties associated with the parameters themselves. The normalized distribution of the simulated data points is represented in the 2D plot in Figure 1 using a color scale and with a black line in the horizontal and vertical histograms. Again, it is possible to observe that at least two groups of informants with different attitudes to the two phenomena under consideration exist.

In Table 5, we summarize the minimum and maximum not-normalized data accessible to and selected by the informants.

**TABLE 5:** Minimum and maximum values of the original (not normalized) G_SCRAM_ and G_OV/VO_ variables.

	**Original minimum values**	**Original maximum values**
G_SCRAM_	min (possible) = −3	max (possible) = +1
	min (survey) = −3	max (survey) = 0
G_OV/VO_	min (possible) = −4	max (possible) = +2
	min (survey) = −4	max (survey) = 0

*For the G_SCRAM_ option, five different options were initially accessible to the informants and three of them are considered in this analysis. Only one of them could be proposed (max = +1) but all of them could be rejected (min = −1 x 3 = −3). Concerning the G_OV/VO_ variable, two sentences were translated and three options were accessible (only two of them are considered in this analysis). Then, for each of these two sentences, only one option could be proposed (max = +1 x 2 = +2), but all of them could be rejected (min = −1 x 4 = −4).*

## Discussion of the Results

In this section, we discuss the results obtained in section “A Statistical Analysis” in detail in order to describe the dimension of micro-variation across Mòcheno varieties.

### Syntax of Embedded Clauses (F1)

Let us begin with F1, which is the position of the finite verb in embedded clauses (OV/VO). In the histogram for F1 in Figure 1 (upper-left panel), the judgments regarding the OV position of the finite verb in embedded clauses for each variety are shown for the German option. In the histogram, we see that the speakers from Fierozzo (red-filled circles and histogram) pattern together coherently in rejecting the OV option (one single peak between 1 and 2). Speakers of the Palù variety (green-filled circles and histogram), by contrast, tended to accept the OV option since the biggest peak for F1_G_ was around 4, even though a small number of speakers rejected it (small secondary peak at around 2). Finally, we see that the distribution of F1_G_ for speakers from Roveda (yellow-filled circles and histogram) exhibit a double peak: a first group of speakers rejected the OV option, whereas another group (F1_G_ peak at around value 3) accepted it, albeit weakly. Let us now consider how the speakers of the three varieties reacted to the second word order proposed, namely the Italian option (VO, Figure 2, upper-left panel). We see that the speakers from Fierozzo (red line) who rejected the OV option consistently accepted the VO word order (F1_I_ peak at around value 5). The same can be said for the majority of speakers from Roveda (yellow line). Finally, the speakers from Palù accepted the Italian VO word order, even though it is clear that it was not their preferred option (F1_I_ peak at around value 3).

### Modal Verbs (F3)

Let us now consider the judgments concerning the possibility of having the German *Satzklammer* together with the Italian linear VO word order in main clauses featuring a modal verb. Let us consider the histogram for F3 in Figure 1 (upper-right panel); that is, the acceptability of the *Satzklammer* in sentences with a modal verb. We see that the F3_G_ data distribution peaks around 4.5 for the Palù variety (green data points and histogram) without significant secondary peaks, and with a very limited deviation from the average value by individual informants. This translates to complete coherency of the informants in accepting the German word order in this case. Since the acceptability rate expressed by the informants from Palù for the Italian word order in the same phenomenon, (F3_I_) peaked at around 3.5 (upper-right panel of Figure 2); thus, we can safely say that, in the Palù variety, OV word order is slightly preferred in this context (which would confirm the preliminary results of the fieldwork conducted in the first phase of the survey discussed in this paper – see section “Methodology”).

Let us consider the other two varieties. For Fierozzo (red data points and histograms), in Figure 1 we see that, unlike in the Palù variety, the data distribution is particularly broad, with F3_G_ presenting two peaks, one at around 1.5 (completely rejected) and one at around 3.5 (weakly accepted). This shows that the German *Satzklammer* option was considered to be either ungrammatical or acceptable by the speakers of this variety, but was not considered to be perfect. This correlates with the data shown in the histogram for F3 in Figure 2 (upper-right panel) in which we see that, when confronted with the Italian VO option, the informants from Fierozzo uniformly judged it to be completely grammatical (a single peak of the F3_I_ distribution at around 5). When confronted with the German *Satzklammer*, speakers from Roveda (yellow data points and histograms in Figure 1, upper-right panel) were distributed broadly, with some informants rejecting the German option (F3_G_ peak located at ∼2.0), and others accepting it (F3_G_ peak at ∼4.5).

Nonetheless, all the speakers consistently judged the Italian option to be the best one (one single peak of F3_I_ at around 5, Figure 2, upper-right panel).

### Syntax of Negation (F2)

Let us now consider the acceptability of the *Satzklammer* and of the VO word order together with the presence/absence of negative concord with negative elements. Let us first consider the upper-right panel in Figure 1. We can see that speakers from Palù (green data points and histograms) mainly judged the German *Satzklammer* as being perfectly grammatical (peak of F2_G_ at around 5), even though some speakers accepted this option weakly (peak of F2_G_ at around value 3). Looking at the same panel in Figure 2, we discover that the German option was only accepted by speakers from Palù, as the single peak of F2_I_ at around the value of 1.5 shows. This indicates that there was no optionality connected to negation for this variety. Let us now consider the Fierozzo variety (red data points and histograms). In Figure 1 (upper-right panel), we see that the German word orders are accepted uniformly (single peak of F2_G_ at around value 4), which correlates with the data in Figure 2 (middle-right panel), showing that the Italian option was rejected (single peak of F2_I_ at value 2). Finally, let us discuss the data for the Roveda variety (yellow line). In Figure 1, we see that there are two peaks for F2_G_ in this case: one at around value 4 and one at around value 2; in other words, one group accepted the German option, whereas another group rejected it. When we look at Figure 2, we see that this correlates with a surprising rejection of the Italian alternatives (peak of F2_I_ at around 2) by all speakers, including those who also rejected the German option.^6^

### Scrambling and OV/VO Word Orders in Main Clauses With a Focused Object (F4)

The presence of two populations within Mòcheno speakers is also confirmed by the acceptability judgments given by informants for the scrambling phenomenon and the distribution of OV/VO word orders in main clauses with a focused light/heavy object. In Figure 4 we see along the GSCRAM axes (upper histogram) a double peak in the distribution (one peak located at +1 and the other at −1), indicating the existence of two main different groups of informants with respect to the scrambling phenomenon: a complete rejection and an average acceptance of the scrambling phenomenon. In this Figure diatopic variation is not considered, but the distribution of informants corresponds to the one sketched above: the German options tend to be accepted by speakers from Palù and rejected (at different degrees) by the speakers of the other varieties.

### Partial Summary

In the preceding sections, we saw that the following strong tendencies emerged from the statistical data. Firstly, the German option was favored in Palù for F1 and F3, and was the only possible alternative in F2. The German option was uniformly rejected for F1 by the speakers from Fierozzo, whereas it was accepted by some of them (two peaks) for F3 and by all of them in the case of F2. The Italian options for F1 and F3 were judged as being perfect by all the speakers from Fierozzo and Roveda, as well as by those who accepted the German option in Figure 1, whereas the speakers of the Palù dialect accepted the Italian word order, but did not judge it as being perfect (the peaks for F1_I_ and F3_I_ are located at ∼3, and should have been at ∼5 if the option were judged to be perfect). For all the three phenomena, informants from Roveda tended to divide themselves into two main groups: one that accepted the German word order, and one that rejected it. This resulted in the double peak distribution that can be seen in all the histograms in Figure 1, including the histogram in the bottom-right panel, in which we sum the results obtained in F1, F2, and F3 for each informant. With the exception of F2_I_, the Italian option was always accepted by all the speakers (including those accepting who accepted the German word order in Figure 1).

These results clearly indicate that there is an implication scale in the acceptability of the German word orders in the phenomena investigated in the paper; that is, negation is the phenomenon in which the German word orders are more likely (or nearly exclusively, see young speakers from Roveda, possibly for independent reasons, see footnote 6) to be the only option, followed by sentences featuring a modal verb. The German word order is less likely to appear in embedded clauses (24).

(24) Negation → modal verbs → embedded clauses

Let us now consider Figure 3, in which we subtracted the results obtained for the Italian option from those obtained for the German option in order to create a scale (Fi_G–I_) in which a more German attitude corresponded to higher (positive) values and a more Italian attitude corresponded to lower (negative) values. A hypothetically perfectly balanced variety, that is a variety in which the German and Italian alternatives are accepted or rejected with similar intensity, would imply that the informants were located at around Fi_G–I_ = 0. This method immediately allowed us to visualize a single variety with regard to the German versus Italian options and to establish the varieties that were considered more German or more Italian for each phenomenon.

In the upper-left panel in Figure 3, for F1_G–I_ we see that Palù is clearly ‘balanced’ in the absolute sense that we expressed above (the peak of the distribution is located at around F1_G–I_∼0). At the same time, the variety of Palù appeared to be more German relative to the other two varieties (that peak at around −3) and should thus be considered to be more Italian than German. For F3_G–I_ (upper-right panel), Palù accepted the German option more strongly; this option was also favored in the other two varieties (peaking at around −2). We still see relative trends similar to those which we described previously for F1_G–I_, but with a general shift of all the informants toward higher (more German) values. Due to this shift, for F3_G–I_, the variant from Palù can be described as more German not only in a relative sense, that is respective to the other varieties (for which the distributions peak at F3_G–I_∼2), but also in an absolute sense (the majority of the informants from Palù were located at F3_G–I_ > 0, with the distribution peaking at F3_G–I_∼0.5). Finally, for F2_G–I_, in the upper-right panel we show that all varieties patterned in very closely, showing a peak at around +3 (Palù) or +2 (Fierozzo and Roveda) – thus adhering definitively to the German option in an absolute sense. Again, given the higher average value of F3_G–I_, the Palù variety is still more German than are the other two.

The bottom-left histogram in Figure 3 allows us to summarize the results described here. When summing the results obtained for the three phenomena for the German option (F1_G_+F2_G_+F3_G_), and subtracting the sum of the results obtained for the Italian option for the same phenomena (F1_I_+F2_I_+F3_I_), we find that the variant of Palù can be considered to be more German than Italian (<F_G–I_ > ∼ 1.0), while the other two variants are more Italian than German (<F_G–I_ > ∼ −1.0).

In particular, the three varieties behave as follows with regard to the German options (see Grewendorf and Poletto, 2005 for data from Cimbrian and Sappadino showing that negative clauses are a conservative environment for OV word orders):

Palù: all phenomena

Fierozzo and Roveda: negation (strong acceptance) → modal (weak acceptance+rejection) → embedded (weak acceptance+rejection)

Crucially, if informants preferred the German option over the Italian one for a given phenomenon, they would also do so with regard to the other phenomena; that is, the value F_G–I_ for ‘Germanicity’ (or ‘Italianicity’) for each individual informant tended to persist in the three different phenomena. In other words, when an informant (or an entire variety) was more ‘German’ than another, based on the values obtained for a single phenomenon, s/he would probably also consider the other phenomena in the same way. This observation is demonstrated by the linear fits that we computed for the three x, y combinations of the phenomena shown in Figure 3, and by the linear analog fit that we computed in Figure 4. In all these cases, we obtained direct, positive correlations amongst the single combinations of the phenomena, with *p*-values that were always lower than 2%.

### On the Role of Diastratic Variation

The last aspect that needs to be discussed is the extent to which the data presented in Figures 1, 2 depend on age; that is, to what extent is there an effect in the observed variation internal to single varieties that is connected to the age of the informants? This has been investigated by considering the average value obtained by the informants for the three different phenomena: (F1+F2+F3)/3. This average is shown for the German option (F_G_), the Italian option (F_I_) and the German-Italian option (F_G–I_) in the bottom-right panels of Figures 1–3, respectively. Let us consider first the bottom-right panel in Figure 1. We see that no effect of age can be detected for Palù (green points), since the speakers were spread uniformly across a range from F_G_∼3 to ∼4.5, irrespective of their ages. The same can be said for Fierozzo (red points), since the speakers in this case were spread uniformly across a range from F_G_∼1.5 to ∼3.5, irrespective of their ages. The speakers from Roveda, by contrast, exhibited internal diastratic variation, since all but one of the younger speakers were located at values of F_G_ lower than 2, whereas elderly speakers (older than 40) were located between F_G_∼3 2 and 4.

When, we consider the acceptability of the Italian versions proposed for phenomena F1, F2, and F3 in Figure 2 (bottom-right panel), once again, we see that no consistent effect of age can be detected: Speakers across all age groups from Palù weakly (F_I_∼3) accepted the Italian word orders, whereas the speakers of the other varieties (F_I_ > 3) accepted them strongly.

If, we consider the average difference between the values obtained for the German and the Italian options (F_G–I_), we see that all the speakers from Palù were always spread between F_G–I_∼0 and F_G–I_∼2 without significant influence of age, whereas speakers from Fierozzo are all found in the area below 0 without clear influence of age, which indicates that they were all consistently ‘less’ German than were speakers from Palù. Instead, diastratic variation was found in Roveda (yellow points), since the younger speakers patterned with the younger speakers from Fierozzo (F_G–I_∼−2), whereas for the elderly speakers, F_G–I_ is located between +1 and −1 (as an average between the groups from Fierozzo and Palù).

### Theoretical Implications

The data discussed in section “Discussion of the Results” above have important implications for our understanding of syntactic variation, particularly regarding the distribution of OV/VO alternations. Firstly, if we consider F1, F2, and F3 in Figures 1, 2, we see that the availability of intra-speaker variation between a German and an Italian word order is not found across all phenomena and all speakers.

Both OV and VO word orders are only attested in embedded clauses (F1) in speakers of the Palù variety. The *Satzklammer* is accepted (along with VO) with modal verbs (F3) by speakers of the Palù dialect, and only by the subgroup of middle-aged and elderly speakers for Fierozzo and Roveda. Finally, the German word order with a negative element is accepted by all speakers except by the younger speakers from Roveda.

(25)
  (a) OV in embedded clause: Palù only;
  (b) Satzklammer with modal verbs: Palù + Fierozzo, group 1 and Roveda group 1;
  (c) Satzklammer with negation: Palù + Fierozzo groups 1, 2; Roveda group 1.

These data clearly indicate that there are two large populations with regard to intra-speaker variation, namely a population (that mainly corresponds to Palù) exhibiting it in F1 and F3, and a population (that mainly corresponds to Fierozzo) exhibiting it in a more opaque way (only in F3, sentences with modal verbs, and not for all speakers). Roveda exhibits intra-speaker variation for F3, but only for a group of speakers (those older than 40). These fine-grained results allow us to refine Rowley (2003) and Cognola (2013a) sociolinguistic descriptions of syntactic variation across Mòcheno varieties by stating that intra-speaker variation is a prerogative of some Mòcheno varieties, and that Fierozzo and Roveda pattern together in the judgments concerning the Italian options (Figure 2), but not with regard to the German word orders (Figure 1) with the exception of younger speakers. The second result is that intra-speaker variation is not found in the syntax of negation (F2) in any variety; that is, the Italian option is rejected consistently by all speakers in F2.

There are two main findings in this paper which need to be accounted for theoretically. The first is that OV/VO word orders coexist within one and the same language: this means that a classic head-parameter account (see Haegeman, 1991 for the head parameter within the Government and Binding framework, Chomsky and Lasnik, 1993 for its application within the Principle and Parameters framework, and also Haider, 2010 for an analysis of German as an OV language) is not suitable for making sense of the documented variation, since Mòcheno is not coherently OV or VO. The second is that the distribution of OV/VO word orders is not constant across the considered groups and across phenomena, which indicates that the distribution of the competing orders it not uniform (which is incompatible with the hypothesis that variation be fed by competing grammars with opposite settings of the head parameter, as in Kroch, 1989) and thus cannot be fed by a single property of grammar.^7^

In order to account for the observed variation, we start out from Kayne (1994) Antisymmetry theory, according to which languages exhibit the universal base order Specifier – Head – Complement (Universal Base Hypothesis), and all OV word orders are to be derived via leftward movement of verb arguments (see Zwart, 1993; Hinterhölzl, 2006; Biberauer and Sheehan, 2013). For the specific case of Mòcheno we assume, following Cognola (2013b); Casalicchio and Cognola (2018), and Cognola and Moroni (2018), that the trigger for leftward movement leading to OV word order is information structure, more specifically a Focus feature associated with a Focus Functional Projection within the vP periphery (Belletti, 2004; Poletto, 2006). The need to check the Focus feature on lowFocusP in the vP periphery forces the constituent to be focused to move out of VP to Spec, lowFocus and the past participle or the infinite verb form to move to lowFocus° – a movement operation which replicates the V2 rule of the higher phase. VO word order involves the movement of the finite verb to lowFocus° and the absence of XP movement (because no pragmatic features need to be checked). In (27) we show a derivation of an OV main clause (26).


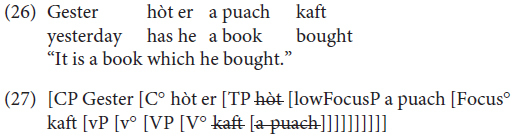


The derivation of main clauses illustrated in (27) should also be assumed for embedded clauses, which, as shown in this paper, allow for OV word order for a subpart of speakers (28a,b). As discussed in Cognola (2015), in fact, OV word order is possible in embedded clauses (along with VO) as long as a topic is extracted (28d), whereas when an operator is long-extracted OV is blocked (28c). We take this to indicate that OV in embedded clauses also involves a focalization in the lower phase which is blocked when an operator is extracted form the lower phase due to cyclic movement (Chomsky, 2001).


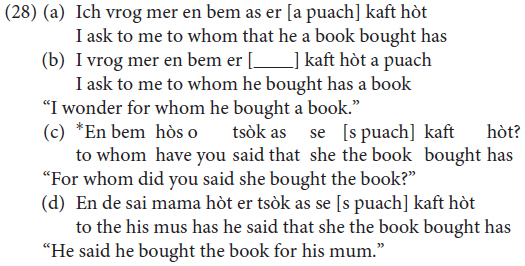


In embedded clauses, the finite verb cannot move to C° (as is typical of V2 languages, see den Besten, 1983) and remains in the lower phase. Therefore, we assume that the linear order past participle – finite verb is derived via movement of the past participle/infinite verb to lowFocus° and of the XP to be focused to Spec, Focus. According to this proposal, the non-finite verb form has to move above the finite verb appearing in v° in embedded clauses.


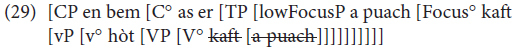


Given the derivations in (27) and (29) how can we account for the differences in the distribution of OV word order across different constructions and different groups of speakers? We propose that this question can be answered building on recent work on Parameter Theory (Roberts, 2012; Biberauer et al., 2014 a.o.; Biberauer and Roberts, 2015, 2016) according to which parameters should be seen as emergent entities and vary in size (Baker, 2008). According to this approach to parameters, a parameter should be understood in a more articulated taxonomy of parameter-types of the kind set out in (30) (taken from Biberauer and Roberts, 2016, p. 260):

(30) For a given value v_i_ of a parametrically variant feature F:
  (a) Macroparameters: all functional heads of the relevant type share v_i_;
  (b) Mesoparameters: all functional heads of a given naturally definable class, e.g., [+V], share v_i_;
  (c) Microparameters: a small subclass of functional heads (e.g., modal auxiliaries) shows v_i_;
  (d) Nanoparameters: one or more individual lexical items is/are specified for v_i_;

The central idea in (30) is that a macroparametric effect obtains when a given property holds for all relevant heads. As one moves downward the hierarchy, the subset of heads characterized by the relevant property increasingly reduces, moving from a natural-class subset of heads (cf. mesoparameter), through a further restricted natural-class subset of heads (cf. microparameter), to a reduced set of lexically specified items (cf. nanoparameter).

Building on Cognola (2013b) analysis of OV word orders in Mòcheno, we propose that the observed data follow from variation in the movement of the past participle to lowFocus° which is parameterized in the way shown in the hierarchy in (31).^8^


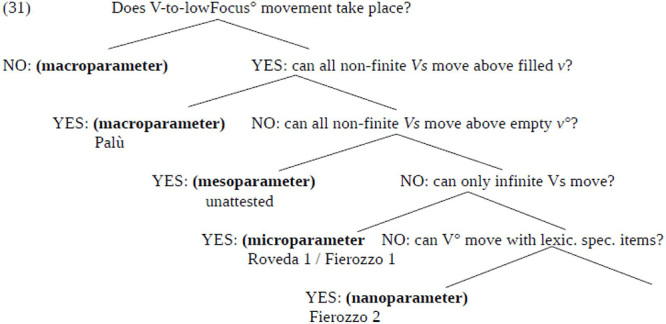


According to the parametric hierarchy given in (31), the fact that OV is grammatical in embedded clauses is configured as a macroparameter affecting the movement of the non-finite verb form above the auxiliary appearing in v°. The positive setting of this parameter allows us to derive the syntactic system of the Palù variety, in which OV is found in all investigated contexts (including embedded clauses). The distribution of OV/VO word orders in sentences featuring a modal verb is captured in terms of a micro-parameter distinguishing the varieties of Fierozzo group 1 and Roveda group 1, whereas the syntax of sentences featuring a negation is a clear example of a nano-parameter, which allows us to distinguish the syntax of Fierozzo group 2. Note, that the mesoparameter distinguishing main clauses from embedded clauses is not attested in the available data, but it is predicted that a subvariety exists exhibiting the parameter on the movement of the finite verb to lowFocus° in this form.

## Conclusion

In this paper, we attempted to provide evidence in favor of an approach to syntactic micro-variation that combines traditional dialectology (fieldwork with informants and sociolinguistic variables) with a statistical analysis (see Van Craenenbroeck et al., 2018 for a similar attempt).

Via an analysis of the judgments of 52 speakers of three Mòcheno varieties with regard to five phenomena assumed to give rise to intra-speaker variation between the German and the Italian word orders, we have demonstrated that there are two large populations: one that exhibits intra-speaker variation and one that does not. These two populations correspond to single varieties (Palù versus Fierozzo) or to populations within a given variety (younger speakers from Roveda pattern with those from Fierozzo, whereas the older ones exhibit intra-speaker variation to a certain extent). Not all the phenomena have been shown to be equally subject to variation: negation (F2) has been shown to exhibit only the German word order for the vast majority of speakers, whereas embedded clauses (F1) have been shown to be the phenomenon in which the German word orders are less generally accepted.

These facts, together with the clear result that age does not play any role in shaping variations, is evidence in favor of the fact that Mòcheno is composed of at least two different populations with different grammars featuring or lacking competing word orders. For the population featuring the two options, it is clear that one of the two (either the German one in the case of Palù or the Italian one for speakers of other varieties, allowing for two orders) is preferred to the other – the challenge is to describe the fine-grained rules that determine the possibility of having the other (possibly marked) option. The results in this paper could have never be achieved using the methodology of traditional linguistic research, in which a reduced number of informants (one per variety, one per age group) is generally considered. This study shows that, in a situation characterized by a complex variation such as that exhibited by Mòcheno, working with a reduced number of informants is dangerous, since individual informants are not necessarily representative of their language variety. The statistical approach allows us to investigate the grammar of individual speakers and of the entire community, thus ensuring that we can describe both the highly specific and the very general correctly.

Based on this fine-grained description of the distribution of OV/VO word orders across different contexts and different groups and the available theoretical account for the derivation of OV word order given by Cognola (2013b), we proposed that the observed variation can be parametrized along the lines of recent developments of Parameter Theory (Roberts, 2012; Biberauer et al., 2014 a.o.). More specifically, we proposed that the movement of the non-finite verb form to lowFocus°, which is responsible for OV, can be captured in terms of a parametric hierarchy. When this movement can take place in all syntactic conditions, including with all non-finite verb forms and when the auxiliary has not moved out of v° to Spec, CP, a macroparametric effect obtains. This is the system instantiated by the Palù variety. The mesoparameter corresponds to a system in which the movement of the non-finite verb form can only be found when v° is empty, i.e., in main declarative clauses. The fact that for Fierozzo group 1 and Roveda group 1 OV word order is accepted with modal verbs follows from a microparamter. Finally, the fact that OV is the only context allowing for OV in all groups (including Fierozzo 2 but excluding Roveda 2) is captured in terms of a nanoparamenter associated with a reduced set of lexically specified items, namely negative constituents.

## Ethics Statement

No ethics approval was required for this study according to Italian law since ethics committees are only needed for clinical trials, see (i) Recepimento delle linee guida dell’Unione europea di buona pratica clinica per la esecuzione delle sperimentazioni cliniche dei medicinali, GU Serie Generale, n. 191 del 18-08-1997 – Suppl. Ordinario n. 162; (ii) Decreto Ministeriale 18 marzo 1998 relativo alle Linee guida di riferimento per l’istituzione e il funzionamento dei Comitati etici pubblicato sulla G.U. n. 122 del 28 maggio 1998; (iii) Decreto del Ministero della Salute 12 maggio 2006 – Requisiti minimi per l’istituzione, l’organizzazione e il funzionamento dei Comitati etici per le sperimentazioni cliniche dei medicinali; (iv) Decreto del Ministero della Salute 8 febbraio 2013 – Criteri per la composizione e il funzionamento dei comitati etici and the Institution’s regulations (ethics committees are only required for clinical studies). The participants’ informed consent was implied through survey completion.

## Author Contributions

FC took responsibility of sections “Introduction” and “The Study” (with the exception of subsection “Population”), subsections “Scrambling and OV/VO Word Orders in Main Clauses With a Focused Object (F4),” “Partial Summary,” “On the Role of Diastratic Variation,” “Theoretical Implications,” and “Conclusion.” IB took responsibility of section “A Statistical Analysis” and subsections “Syntax of Embedded Clauses (F1),” “Modal Verbs (F3),” and “Syntax of Negation (F2).” EM took responsibility of subsection “Population.” FC elaborated the questionnaires and wrote the state-of-the art on the phenomena investigated, the discussion of the results, and the conclusions. IB carried out the statistical analysis and contributed to the discussion of the statistical results. EM carried out the data collection and transcribed the questionnaires.

## Conflict of Interest Statement

The authors declare that the research was conducted in the absence of any commercial or financial relationships that could be construed as a potential conflict of interest.

## Footnotes

1 Standard German must be considered a foreign language for all Mòcheno speakers. The proficiency in German of Mòcheno speakers has never been investigated, except for Ricci Garotti (2013) study which only focuses on school children.

2 *Miga* is a negative adverb (Cinque, 1991) that only appears with a negation (sentence negation *net*), a negative quantifier or a negative quantifier phrase corresponding to German *kein +* NP in Mòcheno. *Bol* is its positive counterpart corresponding to German *ja* which, like German *ja*, is compatible with negation (see Repp, 2013).

3 Note that embedded interrogative clauses are investigated in more detail: this is because this clause type is known to be special with respect to embedded V2, since “the last case in which a Germanic language extends V2 in subordinate clauses is a dependent interrogative” (see Vikner, 1995, cited in Benincà, 2006, p. 69) and Heycock, 2006 on embedded V2 across the Germanic languages. Therefore, this clause type is expected to be the most resistant to embedded V2.

4 In 5/13 sentences a third alternative was given in which two arguments with different discourse status (given-new) appeared within the *Satzklammer*. This alternative was not considered in this paper due to the scarcity of data.

5 The questionnaires completed by three informants were excluded due to evident non-completion/non-understanding of the survey. As a result, the effective number of informants was 52.

6 The speakers from Roveda who rejected both the German and the Italian options were all young speakers. This might be due to independent factors. Firstly, these speakers rejected the use of *nèt* as a constituent negation and only accepted it when used as sentential negation; therefore, they rejected both the *Satzklammer* and the VO word order involving *nèt+XP*. Secondly, these speakers rejected the use of *koa’+NP* as a constituent negation, and substituted the given sentences with a negative quantifier (nobody). Finally, these speakers did not accept the sentences featuring a particle/adverb (such as *bol* or *miga*).

7 The fact that intra-speaker variation is not attested in all groups and in all the phenomena that were investigated clearly speaks in favor of the idea that Mòcheno speakers do not have access to two grammars in competition, one featuring German traits and one featuring Italian traits, but adhere to the rules of the grammar of their variety in which linear German and Italian word orders might be possible, but are not necessarily so. Even speakers who exhibit intra-speaker variation do so in a selective way; that is, negation is not affected by optionality, which again is evidence against the competing grammars hypothesis. These facts also counter the contact hypothesis; in other words, they speak against the availability of two competing grammars as a possible trigger for intra-speaker variation. This is further confirmed by the nature of diastratic variation in Mòcheno. We have seen that age does not play any role in shaping the observed variation, as we do not have a situation in which elderly speakers adhere to a German (more conservative) grammar, whereas younger speakers adhere to an Italian (more innovative) grammar. We consider this to be very strong evidence against the contact hypothesis because younger speakers, who have been exposed to considerably more contact with Italian, pattern with elderly speakers of their variety – this indicates that what appears to play the key role is a speaker belonging to a certain variety rather than the age of the speaker. The only exception is represented by the case of Roveda, in which we see that younger speakers have a different grammar from the grammar of the elderly generation. This, rather than being an effect of direct contact with Italian (which is present for all young Mòcheno speakers, as well as those from Palù), might be due to exposure to the Fierozzo variety from an early an (kindergarten in Fierozzo – see Cognola, 2011). However, more research is needed.

8 We assume that the XPs to be focused move to Spec, lowFocusP independently of the movement of the non-finite verb form to lowFocus°.

## Supplementary Material

DATA SHEET S1 Average answer of each informant, for the questions relative to the German (G, see Figure 1) and the Italian (I, see Figure 2) options about phenomena F1, F2 and F3. In the last three columns, for the same phenomena, we indicate the results of the subtractions between the values obtained for the German and the Italian options (G-I, see Figure 3).

DATA SHEET S2 Average answer of each informant, for the questions relative to scrambling (G_scram_) and OV/VO (G_OV/VO_), as illustrated in Figure 4.
