# Supplementary material for: Inter- vs. Intra-Speaker Variation in Mixed Heritage Syntax: A Statistical Analysis
Source: Front Psychol. 2019 Jul 11;10:1528. doi: 10.3389/fpsyg.2019.01528 (PMC6637972; doi:10.3389/fpsyg.2019.01528)
Supplement: Supplementary file 3 [file Presentation_1.pdf]

NOME E COGNOME: ____

(NB: il nome serve solo a noi, il questionario rimarrà poi anonimo ed a esclusivo uso della ricerca)

ETÁ: ___________

TITOLO DI STUDIO: ____

LINGUE PARLATE: ____

LINGUE PARLATE DAI GENITORI:

MADRE: ____

PADRE: ____

PAESE DI RESIDENZA: ____

VARIETÁ DI MÒCHENO PARLATE DAI GENITORI:

MADRE: _______________________________________

PADRE: _______________________________________

QUANTO FREQUENTEMENTE PARLI MÒCHENO?

____

____

____

Qui di seguito trovi delle frasi identiche nel loro significato che si differenziano però nella loro struttura. Ti chiediamo di dare un giudizio su ogni frase assegnando ad ognuna di esse un voto da 1 (=assolutamente no, non la direi mai) a 5 (assolutamente sì, è una frase che direi)

1 = assolutamente no, non la direi mai
5 = assolutamente sì, è una frase che direi

Nel valutare le frasi ti chiediamo di concentrarti sulla loro struttura (ordine delle parole) e di non considerare, invece, eventuali imperfezioni/errori a livello, per esempio, della scelta delle parole oppure di grafia.

Ti ringraziamo per il tuo aiuto,
buon lavoro
Evelina Molinari, Federica Cognola

DOMANDA 1

a. Derbail as der Mario s puach galesn hòt, pin e gòngen za spiln.
1 □ – 2 □ – 3 □ – 4 □ – 5 □

b. Derbail as der Mario hòt galesn s puach, pin e gòngen za spiln.
1 □ – 2 □ – 3 □ – 4 □ – 5 □

c. Derbail as der Mario hòt s puach galesn, pin e gòngen za spiln.
1 □ – 2 □ – 3 □ – 4 □ – 5 □

Non userei nessuna di queste frasi e direi invece:

____

È possibile togliere AS in una delle frasi sopra? Se sì, in quali?

____

DOMANDA 2

a. S puach derf/kònn se en de sai’ moa’m gem.
1 □ – 2 □ – 3 □ – 4 □ – 5 □

b. S puach derf/kònn se gem en de sai’ moa’m.
1 □ – 2 □ – 3 □ – 4 □ – 5 □

Non userei nessuna di queste frasi e direi invece:

____

DOMANDA 3

a. I hòn nèt koa’ mentsch tsechen nèt.
1 □ – 2 □ – 3 □ – 4 □ – 5 □

b. I hòn koa’ mentsch nèt tsechen nèt.
1 □ – 2 □ – 3 □ – 4 □ – 5 □

Non userei nessuna di queste frasi e direi invece:

____

DOMANDA 4

a. De hom mer tsòk/kein as der Mario s puach galesn hòt.
1 □ – 2 □ – 3 □ – 4 □ – 5 □

b. De hom mer tsòk/kein as der Mario hòt galesn s puach.
1 □ – 2 □ – 3 □ – 4 □ – 5 □

c. De hom mer tsòk/kein as der Mario hòt s puach galesn.
1 □ – 2 □ – 3 □ – 4 □ – 5 □

Non userei nessuna di queste frasi e direi invece:

____

È possibile togliere AS in una delle frasi sopra? Se sì, in quali?

____

DOMANDA 5

a. I hòn nèt koa' mentsch tsechen.
1 □ – 2 □ – 3 □ – 4 □ – 5 □

b. I hòn miga koa' mentsch tsechen.
1 □ – 2 □ – 3 □ – 4 □ – 5 □

c. I hòn bol koa' mentsch tsechen.
1 □ – 2 □ – 3 □ – 4 □ – 5 □

Non userei nessuna di queste frasi e direi invece:

____

DOMANDA 6

a. I denk as der Mario s puach galesn hòt.
1 □ – 2 □ – 3 □ – 4 □ – 5 □

b. I denk as der Mario hòt galesn s puach.
1 □ – 2 □ – 3 □ – 4 □ – 5 □

c. I denk as der Mario hòt s puach galesn.
1 □ – 2 □ – 3 □ – 4 □ – 5 □

Non userei nessuna di queste frasi e direi invece:

____

È possibile togliere AS in una delle frasi sopra? Se sì, in quali?

____

DOMANDA 7

<u>Qualcuno mi chiede</u>:
Bos schellt se kaven en de Maria?

<u>Io rispondo</u>:

a. En de Maria schellt se a puach kaven.
1 □ – 2 □ – 3 □ – 4 □ – 5 □

b. En de Maria schellt se kaven a puach.
1 □ – 2 □ – 3 □ – 4 □ – 5 □

c. De schellt en de Maria a puach kaven.
1 □ – 2 □ – 3 □ – 4 □ – 5 □

Non userei nessuna di queste frasi e direi invece:

____

DOMANDA 8

a. I mua nèt as der Mario s puach galesn hòt.
1 □ – 2 □ – 3 □ – 4 □ – 5 □

b. I mua nèt as der Mario hòt galesn s puach.
1 □ – 2 □ – 3 □ – 4 □ – 5 □

c. I mua nèt as der Mario hòt s puach galesn.
1 □ – 2 □ – 3 □ – 4 □ – 5 □

Non userei nessuna di queste frasi e direi invece:

____

È possibile togliere AS in una delle frasi sopra? Se sì, in quali?

____

DOMANDA 9

a. S tuat mer loat as der Mario de sai òrbet verlourn hòt.
1 □ – 2 □ – 3 □ – 4 □ – 5 □

b. S tuat mer loat as der Mario hòt verlourn de sai òrbet.
1 □ – 2 □ – 3 □ – 4 □ – 5 □

c. S tuat mer loat as der Mario hòt de sai òrbet verlourn .
1 □ – 2 □ – 3 □ – 4 □ – 5 □

Non userei nessuna di queste frasi e direi invece:

____

È possibile togliere AS in una delle frasi sopra? Se sì, in quali?

____

DOMANDA 10

a. I hòn koa' hunt tsechen, lai an schotn.
1 □ – 2 □ – 3 □ – 4 □ – 5 □

b. I hòn koa' hunt tsechen, ober an schotn.
1 □ – 2 □ – 3 □ – 4 □ – 5 □

c. I hòn koa' hunt tsechen, ma an schotn.
1 □ – 2 □ – 3 □ – 4 □ – 5 □

Non userei nessuna di queste frasi e direi invece:

____

DOMANDA 11

a. I hòn tsechen as der Mario s puach galesn hòt.
1 □ – 2 □ – 3 □ – 4 □ – 5 □

b. I hòn tsechen as der Mario hòt galesn s puach.
1 □ – 2 □ – 3 □ – 4 □ – 5 □

c. I hòn tsechen as der Mario hòt s puach galesn.
1 □ – 2 □ – 3 □ – 4 □ – 5 □

Non userei nessuna di queste frasi e direi invece:

____

È possibile togliere AS in una delle frasi sopra? Se sì, in quali?

____

DOMANDA 12

a.Benn as o/de ka Persn gòngen pist, hòs o/hòst kaft s puach.
1 □ – 2 □ – 3 □ – 4 □ – 5 □

b. Benn as o/de pist gòngen ka Persn, hòs o/hòst kaft s puach.
1 □ – 2 □ – 3 □ – 4 □ – 5 □

c. Benn as o/de pist ka Persn gòngen, hòs o/hòst kaft s puach.
1 □ – 2 □ – 3 □ – 4 □ – 5 □

Non userei nessuna di queste frasi e direi invece:

____

È possibile togliere AS in una delle frasi sopra? Se sì, in quali?

____

DOMANDA 13

a. Derno as de mama s puach kaft hòt, hòt se se garauchen/garichen tsechen.
1 □ – 2 □ – 3 □ – 4 □ – 5 □

b. Derno as de mama hòt kaft s puach, hòt se se garauchen/garichen tsechen.
1 □ – 2 □ – 3 □ – 4 □ – 5 □

c. Derno as de mama hòt s puach kaft, hòt se se garauchen/garichen tsechen.
1 □ – 2 □ – 3 □ – 4 □ – 5 □

Non userei nessuna di queste frasi e direi invece:

____

È possibile togliere AS in una delle frasi sopra? Se sì, in quali?

____

DOMANDA 14

a. Ber schellt òlbe s haus putzn?

1 □ – 2 □ – 3 □ – 4 □ – 5 □

b. Ber schellt òlbe putzn s haus?

1 □ – 2 □ – 3 □ – 4 □ – 5 □

Non userei nessuna di queste frasi e direi invece:

____

DOMANDA 15

a. I hòn nèt bai' trunken.

1 □ – 2 □ – 3 □ – 4 □ – 5 □

b. I hòn trunken nèt bai'.

1 □ – 2 □ – 3 □ – 4 □ – 5 □

Non userei nessuna di queste frasi e direi invece:

____

DOMANDA 16

a. An iats vort as mer der Mario a puach kaft hòt, hòt er pfalt.

1 □ – 2 □ – 3 □ – 4 □ – 5 □

b. An iats vort as der Mario hòt mer kaft a puach, hòt er pfalt.

1 □ – 2 □ – 3 □ – 4 □ – 5 □

c. An iats vort as der Mario hòt mer a puach kaft, hòt er pfalt.

1 □ – 2 □ – 3 □ – 4 □ – 5 □

Non userei nessuna di queste frasi e direi invece:

____

È possibile togliere AS in una delle frasi sopra? Se sì, in quali?

____

DOMANDA 17

Qualuno mi chiede:
Bos tuat er èssn der Mario hait?

Io rispondo:

a. Hait der Mario tuat sushi èssn.
1 □ – 2 □ – 3 □ – 4 □ – 5 □

b. Hait der Mario tuat èssn sushi.
1 □ – 2 □ – 3 □ – 4 □ – 5 □

c. Hait tuat der Mario sushi èssn.
1 □ – 2 □ – 3 □ – 4 □ – 5 □

Non userei nessuna di queste frasi e direi invece:

____

DOMANDA 18

a. I hòn en galeart abia as der Mario der/en kas gamòcht hòt.
1 □ – 2 □ – 3 □ – 4 □ – 5 □

b. I hòn en galeart abia as der Mario hòt gamòcht der/en kas.
1 □ – 2 □ – 3 □ – 4 □ – 5 □

c. I hòn en galeart abia as der Mario hòt der/en kas gamòcht.
1 □ – 2 □ – 3 □ – 4 □ – 5 □

Non userei nessuna di queste frasi e direi invece:

____

È possibile togliere AS in una delle frasi sopra? Se sì, in quali?

____

DOMANDA 19

Qualcuno mi chiede:
Bos kònn se kaven en de Maria?

Io rispondo:

a. En de Maria kònn se a puach kaven.
1 □ – 2 □ – 3 □ – 4 □ – 5 □

b. En de Maria kònn se kaven a puach.

1 □ – 2 □ – 3 □ – 4 □ – 5 □

c. De kònn en de Maria a puach kaven.

1 □ – 2 □ – 3 □ – 4 □ – 5 □

Non userei nessuna di queste frasi e direi invece:

____

DOMANDA 20

a. I pin spat kemmen vavai as de mama de koriara verlourn hòt.

1 □ – 2 □ – 3 □ – 4 □ – 5 □

b. I pin spat kemmen vavai as de mama hòt verlourn de koriara.

1 □ – 2 □ – 3 □ – 4 □ – 5 □

c. I pin spat kemmen vavai as de mama hòt de koriara verlourn.

1 □ – 2 □ – 3 □ – 4 □ – 5 □

Non userei nessuna di queste frasi e direi invece:

____

È possibile togliere AS in una delle frasi sopra? Se sì, in quali?

____

DOMANDA 21

a. I hòn bol nèt pet diar klòfft.

1 □ – 2 □ – 3 □ – 4 □ – 5 □

b. I hòn miga nèt pet diar klòfft.

1 □ – 2 □ – 3 □ – 4 □ – 5 □

c. I hòn nèt miga pet diar klòfft.

1 □ – 2 □ – 3 □ – 4 □ – 5 □

Non userei nessuna di queste frasi e direi invece:

____

DOMANDA 22

Qualcuno mi chiede:
Bos tea' se kaven en de Maria?

Io rispondo:

a. En de Maria tea' se a puach kaven.
1 □ – 2 □ – 3 □ – 4 □ – 5 □

b. En de Maria tea' se kaven a puach.
1 □ – 2 □ – 3 □ – 4 □ – 5 □

c. De tea' en de Maria a puach kaven.
1 □ – 2 □ – 3 □ – 4 □ – 5 □

Non userei nessuna di queste frasi e direi invece:

____

DOMANDA 23

a. Vavai as de mama de koriara verlourn hòt, ist se spat kemmen.
1 □ – 2 □ – 3 □ – 4 □ – 5 □

b. Vavai as de mama hòt verlourn de koriara, ist se spat kemmen.
1 □ – 2 □ – 3 □ – 4 □ – 5 □

c. Vavai as de mama hòt de koriara verlourn, ist se spat kemmen.
1 □ – 2 □ – 3 □ – 4 □ – 5 □

Non userei nessuna di queste frasi e direi invece:

____

È possibile togliere AS in una delle frasi sopra? Se sì, in quali?

____

DOMANDA 24

CONTESTO: Mario arriva tutti i giorni in classe alle 7.30. Oggi è arrivato alle 8.00. I suoi compagni parlano tra di loro e ipotizzano che abbia perso la corriera.

a. Er mias de koriara verlourn hom, vavai as er spat kemmen ist.
1 □ – 2 □ – 3 □ – 4 □ – 5 □

b. Er mias de koriara verlourn hom, vavai as er ist kemmen spat.

1 □ – 2 □ – 3 □ – 4 □ – 5 □

c. Er mias de koriara verlourn hom, vavai as er ist spat kemmen.

1 □ – 2 □ – 3 □ – 4 □ – 5 □

Non userei nessuna di queste frasi e direi invece:

____

È possibile togliere AS in una delle frasi sopra? Se sì, in quali?

____

DOMANDA 25

Qualcuno mi chiede:
Der Nane kimmp nèt?

Io rispondo:

a. Na, er kimmp nèt.

□ – 2 □ – 3 □ – 4 □ – 5 □

a. Jo, er kimmp nèt.

1 □ – 2 □ – 3 □ – 4 □ – 5 □

Non userei nessuna di queste frasi e direi invece:

____

DOMANDA 26

CONTESTO: Mario arriva tutti i giorni in classe alle 7.30. Oggi è arrivato alle 8.00. I suoi compagni parlano tra di loro e ipotizzano che abbia perso la corriera.

a. Vavai as er spat kemmen ist, mias er de koriara verlourn hom.

1 □ – 2 □ – 3 □ – 4 □ – 5 □

b. Vavai as er ist kemmen spat, mias er de koriara verlourn hom.

1 □ – 2 □ – 3 □ – 4 □ – 5 □

c. Vavai as er ist spat kemmen, mias er de koriara verlourn hom.

1 □ – 2 □ – 3 □ – 4 □ – 5 □

Non userei nessuna di queste frasi e direi invece:

____

È possibile togliere AS in una delle frasi sopra? Se sì, in quali?

____

DOMANDA 27

a. I boas nèt vavai as er der sushi gèssn hòt.
1 □ – 2 □ – 3 □ – 4 □ – 5 □

b. I boas nèt vavai as er hòt gèssn der sushi.
1 □ – 2 □ – 3 □ – 4 □ – 5 □

c. I boas nèt vavai as er hòt der sushi gèssn.
1 □ – 2 □ – 3 □ – 4 □ – 5 □

Non userei nessuna di queste frasi e direi invece:

____

È possibile togliere AS in una delle frasi sopra? Se sì, in quali?

____

DOMANDA 28

a. Vavai as er der sushi gèssn hòt, boas e nèt.
1 □ – 2 □ – 3 □ – 4 □ – 5 □

b. Vavai as er hòt gèssn der sushi, boas e nèt.
1 □ – 2 □ – 3 □ – 4 □ – 5 □

c. Vavai as er hòt der sushi gèssn, boas e nèt.
1 □ – 2 □ – 3 □ – 4 □ – 5 □

Non userei nessuna di queste frasi e direi invece:

____

È possibile togliere AS in una delle frasi sopra? Se sì, in quali?

____

DOMANDA 29

a. Der Mario hòt mer pfrok bos as de Rita ver de mama kaft hòt.
1 □ – 2 □ – 3 □ – 4 □ – 5 □

b. Der Mario hòt mer pfrok bos as de Rita hòt kaft ver de mama.

1 □ – 2 □ – 3 □ – 4 □ – 5 □

c. Der Mario hòt mer pfrok bos as de Rita hòt ver de mama kaft.

1 □ – 2 □ – 3 □ – 4 □ – 5 □

Non userei nessuna di queste frasi e direi invece:

____

È possibile togliere AS in una delle frasi sopra? Se sì, in quali?

____

DOMANDA 30

a. Der Mario hòt mer pfrok, ber as de Maria ka Persn tsechen hòt.

1 □ – 2 □ – 3 □ – 4 □ – 5 □

b. Der Mario hòt mer pfrok, ber as de Maria hòt tsechen ka Persn.

1 □ – 2 □ – 3 □ – 4 □ – 5 □

c. Der Mario hòt mer pfrok, ber as de Maria hòt ka Persn tsechen.

1 □ – 2 □ – 3 □ – 4 □ – 5 □

Non userei nessuna di queste frasi e direi invece:

____

È possibile togliere AS in una delle frasi sopra? Se sì, in quali?

____

DOMANDA 31

a. De mama bill de sai' schbester pakemmen.

1 □ – 2 □ – 3 □ – 4 □ – 5 □

b. De mama bill pakemmen de sai' schbester.

1 □ – 2 □ – 3 □ – 4 □ – 5 □

Non userei nessuna di queste frasi e direi invece:

____

DOMANDA 32

a. I boas nèt biavle lait as ka Persn kemmen sai'.

1 □ – 2 □ – 3 □ – 4 □ – 5 □

b. I boas nèt biavle lait as sai' kemmen ka Persn.

1 □ – 2 □ – 3 □ – 4 □ – 5 □

c. I boas nèt biavle lait as sai' ka Persn kemmen.

1 □ – 2 □ – 3 □ – 4 □ – 5 □

Non userei nessuna di queste frasi e direi invece:

____

È possibile togliere AS in una delle frasi sopra? Se sì, in quali?

____

DOMANDA 33

<u>CONTESTO</u>: C'è stato un furto in una casa. Il ladro è entrato dalla finestra, che ora è rotta. Il proprietario dell'appartamento dice:

a. I boas nèt benn as der diab der/en bolkèt prochen hòt.

1 □ – 2 □ – 3 □ – 4 □ – 5 □

b. I boas nèt benn as der diab hòt prochen der/en bòlket.

1 □ – 2 □ – 3 □ – 4 □ – 5 □

c. I boas nèt benn as der diab hòt der/en bòlket prochen.

1 □ – 2 □ – 3 □ – 4 □ – 5 □

Non userei nessuna di queste frasi e direi invece:

____

È possibile togliere AS in una delle frasi sopra? Se sì, in quali?

____

DOMANDA 34

<u>Qualcuno mi chiede</u>:
Bos bill er èssn der Mario hait?

<u>Io rispondo</u>:

a. Hait der Mario bill sushi èssn.

1 □ – 2 □ – 3 □ – 4 □ – 5 □

b. Hait der Mario bill èssn sushi.

1 □ – 2 □ – 3 □ – 4 □ – 5 □

c. Hait bill der Mario sushi èssn.

1 □ – 2 □ – 3 □ – 4 □ – 5 □

Non userei nessuna di queste frasi e direi invece:

____

DOMANDA 35

<u>CONTESTO</u>: Il bambino ha mangiato solo metà della pappa che aveva nel piatto. La mamma pensa:

a. I vrog mer, benn as s kinn ganua' muas gèssn hòt.

1 □ – 2 □ – 3 □ – 4 □ – 5 □

b. I vrog mer, benn as s kinn hòt gèssn ganua' muas.

1 □ – 2 □ – 3 □ – 4 □ – 5 □

c. I vrog mer, benn as s kinn hòt ganua' muas gèssn.

1 □ – 2 □ – 3 □ – 4 □ – 5 □

Non userei nessuna di queste frasi e direi invece:

____

È possibile togliere AS in una delle frasi sopra? Se sì, in quali?

____

DOMANDA 36

a. De hòt mer pfrok, abia as de Laura ka Persn gòngen ist.

1 □ – 2 □ – 3 □ – 4 □ – 5 □

b. De hòt mer pfrok, abia as de Laura ist gòngen ka Persn.

1 □ – 2 □ – 3 □ – 4 □ – 5 □

c. De hòt mer pfrok, abia as de Laura ist ka Persn gòngen.

1 □ – 2 □ – 3 □ – 4 □ – 5 □

Non userei nessuna di queste frasi e direi invece:

____

È possibile togliere AS in una delle frasi sopra? Se sì, in quali?

____

DOMANDA 37

a. Er hòt mer pfrok, en bem as de Agnese s gèlt gem hòt.
1 □ – 2 □ – 3 □ – 4 □ – 5 □

b. Er hòt mer pfrok, en bem as de Agnese hòt gem s gèlt.
□ – 2 □ – 3 □ – 4 □ – 5 □

b. Er hòt mer pfrok, en bem as de Agnese hòt s gèlt gem.
1 □ – 2 □ – 3 □ – 4 □ – 5 □

Non userei nessuna di queste frasi e direi invece:

____

È possibile togliere AS in una delle frasi sopra? Se sì, in quali?

____

DOMANDA 38

a. Er hòt koa’ bai’ trunken.
1 □ – 2 □ – 3 □ – 4 □ – 5 □

b. I hòn trunken koa’ bai’.
1 □ – 2 □ – 3 □ – 4 □ – 5 □

Non userei nessuna di queste frasi e direi invece:

____

DOMANDA 39

a. I vrog mer, bos ver a mentsch as de doin beirter song mechat.
1 □ – 2 □ – 3 □ – 4 □ – 5 □

b. I vrog mer, bos ver a mentsch as mechat song de doin beirter.
1 □ – 2 □ – 3 □ – 4 □ – 5 □

c. I vrog mer, bos ver a mentsch as mechat de doin beirter song.
1 □ – 2 □ – 3 □ – 4 □ – 5 □

Non userei nessuna di queste frasi e direi invece:

____

È possibile togliere AS in una delle frasi sopra? Se sì, in quali?

____

DOMANDA 40

a. Er hòt mer pfrok, s beil puach as de Maria ver de mama kaft hòt.
1 □ – 2 □ – 3 □ – 4 □ – 5 □

b. Er hòt mer pfrok, s beil puach as de Maria hòt kaft ver de mama.
1 □ – 2 □ – 3 □ – 4 □ – 5 □

c. Er hòt mer pfrok, s beil puach as de Maria hòt ver de mama kaft.
1 □ – 2 □ – 3 □ – 4 □ – 5 □

Non userei nessuna di queste frasi e direi invece:

____

È possibile togliere AS in una delle frasi sopra? Se sì, in quali?

____

DOMANDA 41

a. I boas nèt bou as der Rudolf s doi puach kaven mechat.
1 □ – 2 □ – 3 □ – 4 □ – 5 □

b. I boas nèt bou as der Rudolf mechat kaven s doi puach.
1 □ – 2 □ – 3 □ – 4 □ – 5 □

c. I boas nèt bou as der Rudolf mechat s doi puach kaven.
1 □ – 2 □ – 3 □ – 4 □ – 5 □

Non userei nessuna di queste frasi e direi invece:

____

È possibile togliere AS in una delle frasi sopra? Se sì, in quali?

____

DOMANDA 42

a. Benn as s kinn de gònze pasta gèssn hòt, bart s spiln meing.
1 □ – 2 □ – 3 □ – 4 □ – 5 □

b. Benn as s kinn hòt gèssn de gònze pasta, bart s spiln meing.
1 □ – 2 □ – 3 □ – 4 □ – 5 □

c. Benn as s kinn hòt de gònze pasta gèssn, bart s spiln meing.

1 □ – 2 □ – 3 □ – 4 □ – 5 □

Non userei nessuna di queste frasi e direi invece:

____

È possibile togliere AS in una delle frasi sopra? Se sì, in quali?

____

DOMANDA 43

a. De mama tuat de sai' schbester pakemmen.

1 □ – 2 □ – 3 □ – 4 □ – 5 □

b. De mama tuat pakemmen de sai' schbester.

1 □ – 2 □ – 3 □ – 4 □ – 5 □

Non userei nessuna di queste frasi e direi invece:

____

DOMANDA 44

a. Benn as de Maria aa s puach kaft hat, hat se nèt garift de compete ver hait.

1 □ – 2 □ – 3 □ – 4 □ – 5 □

b. Benn as de Maria aa hat kaft s puach, hat se nèt garift de compete ver hait.

1 □ – 2 □ – 3 □ – 4 □ – 5 □

c. Benn as de Maria aa hat s puach kaft, hat se nèt garift de compete ver hait.

1 □ – 2 □ – 3 □ – 4 □ – 5 □

Non userei nessuna di queste frasi e direi invece:

____

È possibile togliere AS in una delle frasi sopra? Se sì, in quali?

____

DOMANDA 45

a. Du hòst nèt koa’ puach galesn.
1 □ – 2 □ – 3 □ – 4 □ – 5 □

b. Du hòst koa’ puach nèt galesn.
1 □ – 2 □ – 3 □ – 4 □ – 5 □

c. Du hòst koa’ puach galesn nèt.
1 □ – 2 □ – 3 □ – 4 □ – 5 □

Non userei nessuna di queste frasi e direi invece:

____

DOMANDA 46

a. Benn as de Maria aa ver en esam studiert hat, kannat se nèt ompòrt gem en de doi vrog.
1 □ – 2 □ – 3 □ – 4 □ – 5 □

b. Benn as de Maria aa hat studiert ver en esame, kannat se nèt ompòrt gem en de doi vrog.
1 □ – 2 □ – 3 □ – 4 □ – 5 □

c. Benn as de Maria aa hat ver en esam studiert, kannat se nèt ompòrt gem en de doi vrog.
1 □ – 2 □ – 3 □ – 4 □ – 5 □

Non userei nessuna di queste frasi e direi invece:

____

È possibile togliere AS in una delle frasi sopra? Se sì, in quali?

____

DOMANDA 47

b. Er ist asou stuf gaben za lesn, as er s puach vòrt gaborven hat.
1 □ – 2 □ – 3 □ – 4 □ – 5 □

b. Er ist asou stuf gaben za lesn, as er hat vòrt gaborven s puach .
1 □ – 2 □ – 3 □ – 4 □ – 5 □

c. Er ist asou stuf gaben za lesn, as er hat s puach vòrt gaborven.
1 □ – 2 □ – 3 □ – 4 □ – 5 □

Non userei nessuna di queste frasi e direi invece:

____

È possibile togliere AS in una delle frasi sopra? Se sì, in quali?

___

DOMANDA 48

a. Er klòfft abia benn as er er aloa' s puach galesn hat.
1 □ – 2 □ – 3 □ – 4 □ – 5 □

b. Er klòfft abia benn as er er aloa' hat galesn s puach.
1 □ – 2 □ – 3 □ – 4 □ – 5 □

c. Er klòfft abia benn as er er aloa' hat s puach galesn.
1 □ – 2 □ – 3 □ – 4 □ – 5 □

Non userei nessuna di queste frasi e direi invece:

___

È possibile togliere AS in una delle frasi sopra? Se sì, in quali?

___

DOMANDA 49

a. De sai' gòngen a'ne as der Mario de dokumentn zaruck gem hòt en.
1 □ – 2 □ – 3 □ – 4 □ – 5 □

b. De sai' gòngen a'ne as der Mario hòt en zaruck gem de dokumentn.
1 □ – 2 □ – 3 □ – 4 □ – 5 □

c. De sai' gòngen a'ne as der Mario hòt en de dokumentn zaruckgem.
1 □ – 2 □ – 3 □ – 4 □ – 5 □

Non userei nessuna di queste frasi e direi invece:

___

È possibile togliere AS in una delle frasi sopra? Se sì, in quali?

___

DOMANDA 50

a. I hòn nèt an hunt tsechen, lai an schotn.
1 □ – 2 □ – 3 □ – 4 □ – 5 □

b. I hòn nèt an hunt tsechen, ober an schotn.
1 □ – 2 □ – 3 □ – 4 □ – 5 □

c. I hòn nèt an hunt tsechen, ma an schotn.

1 □ – 2 □ – 3 □ – 4 □ – 5 □

Non userei nessuna di queste frasi e direi invece:

____

DOMANDA 51

a. Òlla hom sa nen tsechen der Luca, aus de sèlln as en haus gaben sai'.

1 □ – 2 □ – 3 □ – 4 □ – 5 □

b. Òlla hom sa nen tsechen der Luca, aus de sèlln as sai' gaben en haus.

1 □ – 2 □ – 3 □ – 4 □ – 5 □

c. Òlla hom sa nen tsechen der Luca, aus de sèlln as sai' en haus gaben.

1 □ – 2 □ – 3 □ – 4 □ – 5 □

Non userei nessuna di queste frasi e direi invece:

____

È possibile togliere AS in una delle frasi sopra? Se sì, in quali?

____

DOMANDA 52

a. Ber tuat òlbe s haus putzn?

1 □ – 2 □ – 3 □ – 4 □ – 5 □

b. Ber tuat òlbe putzn s haus?

1 □ – 2 □ – 3 □ – 4 □ – 5 □

Non userei nessuna di queste frasi e direi invece:

____

DOMANDA 53

a. Der Mario, as/bo pet en sai' baib kemmen ist, ist der mai' kusi'.

1 □ – 2 □ – 3 □ – 4 □ – 5 □

b. Der Mario, as/bo ist kemmen pet en sai' baib, ist der mai' kusi'.

1 □ – 2 □ – 3 □ – 4 □ – 5 □

c. Der Mario, as/bo ist pet en sai' baib kemmen, ist der mai' kusi'.

1 □ – 2 □ – 3 □ – 4 □ – 5 □

Non userei nessuna di queste frasi e direi invece:

____

È possibile togliere AS in una delle frasi sopra? Se sì, in quali?

____

DOMANDA 54

a. I hòn s kinn tsechen, as/bo o pet de Maria pakemmp hòst.

1 □ – 2 □ – 3 □ – 4 □ – 5 □

b. I hòn tsechen s kinn, as/bo de hòst pakemmp pet de Maria.

1 □ – 2 □ – 3 □ – 4 □ – 5 □

c. I hòn tsechen s kinn, as/bo o/de hòst pet de Maria pakemmp.

1 □ – 2 □ – 3 □ – 4 □ – 5 □

Non userei nessuna di queste frasi e direi invece:

____

È possibile togliere AS in una delle frasi sopra? Se sì, in quali?

____

DOMANDA 55

a. I hòn bol nèt miga pet diar klòfft nèt.

1 □ – 2 □ – 3 □ – 4 □ – 5 □

b. I hòn bol miga nèt pet diar klòfft nèt.

1 □ – 2 □ – 3 □ – 4 □ – 5 □

c. I hòn miga bol nèt pet diar klòfft nèt.

1 □ – 2 □ – 3 □ – 4 □ – 5 □

Non userei nessuna di queste frasi e direi invece:

____

DOMANDA 56

a. I denk as der Mario s puach galesn hòt.

1 □ – 2 □ – 3 □ – 4 □ – 5 □

b. I denk as der Mario hòt galesn s puach.

1 □ – 2 □ – 3 □ – 4 □ – 5 □

c. I denk as der Mario hòt s puach galesn.

1 □ – 2 □ – 3 □ – 4 □ – 5 □

Non userei nessuna di queste frasi e direi invece:

____

È possibile togliere AS in una delle frasi sopra? Se sì, in quali?

____

DOMANDA 57

a. I hòn pakemmp s sèll kinn as du mer kan Nane kontart (gahòp) hòst.

1 □ – 2 □ – 3 □ – 4 □ – 5 □

b. I hòn pakemmp s sèll kinn as du hòst mer (gahòp) kontart kan Nane.

1 □ – 2 □ – 3 □ – 4 □ – 5 □

c. I hòn pakemmp s sèll kinn as du hòst mer kan Nane (gahòp) kontart.

1 □ – 2 □ – 3 □ – 4 □ – 5 □

Non userei nessuna di queste frasi e direi invece:

____

È possibile togliere AS in una delle frasi sopra? Se sì, in quali?

____

DOMANDA 58

a. S puach mu se en de sai’ moa’m gem.

1 □ – 2 □ – 3 □ – 4 □ – 5 □

b. S puach mu se gem en de sai’ moa’m.

1 □ – 2 □ – 3 □ – 4 □ – 5 □

Non userei nessuna di queste frasi e direi invece:

____

DOMANDA 59

a. I hòn nèt bol pet diar klòfft.

1 □ – 2 □ – 3 □ – 4 □ – 5 □

b. I hòn bol nèt pet diar klòfft.

1 □ – 2 □ – 3 □ – 4 □ – 5 □

Non userei nessuna di queste frasi e direi invece:

____

DOMANDA 60

a. S puach tuat se en de sai’ moa’m gem.

1 □ – 2 □ – 3 □ – 4 □ – 5 □

b. S puach tuat se gem en de sai’ moa’m.

1 □ – 2 □ – 3 □ – 4 □ – 5 □

Non userei nessuna di queste frasi e direi invece:

____

DOMANDA 61

<u>Qualcuno mi chiede</u>:
Der Nane kimmp nèt?

<u>Io rispondo</u>:

a. Na, er kimmt bol

1 □ – 2 □ – 3 □ – 4 □ – 5 □

d. Jo, er kimmp bol.

1 □ – 2 □ – 3 □ – 4 □ – 5 □

Non userei nessuna di queste frasi e direi invece:

____

DOMANDA 62

a. Ber mias òlbe s haus putzn?

1 □ – 2 □ – 3 □ – 4 □ – 5 □

b. Ber mias òlbe putzn s haus?

1 □ – 2 □ – 3 □ – 4 □ – 5 □

Non userei nessuna di queste frasi e direi invece:

____
